# The importance of street trees to urban avifauna

**DOI:** 10.1002/eap.2149

**Published:** 2020-06-11

**Authors:** Eric M. Wood, Sevan Esaian

**Affiliations:** ^1^ Department of Biological Sciences California State University Los Angeles 5151 State University Drive Los Angeles California 90032 USA; ^2^ Ecology, Evolution, and Marine Biology University of California Santa Barbara Santa Barbara California 93106‐9620 USA

**Keywords:** bird, California, foraging behavior, Los Angeles, migratory, native vegetation, nonnative vegetation, socioeconomic, urban forest, wildlife

## Abstract

Street trees are public resources planted in a municipality’s right‐of‐way and are a considerable component of urban forests throughout the world. Street trees provide numerous benefits to people. However, many metropolitan areas have a poor understanding of the value of street trees to wildlife, which presents a gap in our knowledge of conservation in urban ecosystems. Greater Los Angeles (LA) is a global city harboring one of the most diverse and extensive urban forests on the planet. The vast majority of the urban forest is nonnative in geographic origin, planted throughout LA following the influx of irrigated water in the early 1900s. In addition to its extensive urban forest, LA is home to a high diversity of birds, which utilize the metropolis throughout the annual cycle. The cover of the urban forest, and likely street trees, varies dramatically across a socioeconomic gradient. However, it is unknown how this variability influences avian communities. To understand the importance of street trees to urban avifauna, we documented foraging behavior by birds on native and nonnative street trees across a socioeconomic gradient throughout LA. Affluent communities harbored a unique composition of street trees, including denser and larger trees than lower‐income communities, which in turn, attracted nearly five times the density of feeding birds. Foraging birds strongly preferred two native street‐tree species as feeding substrates, the coast live oak (*Quercus agrifolia*) and the California sycamore (*Platanus racemosa*), and a handful of nonnative tree species, including the Chinese elm (*Ulmus parvifolia*), the carrotwood (*Cupaniopsis anacardioides*), and the southern live oak (*Quercus virginiana*), in greater proportion than their availability throughout the cityscape (two to three times their availability). Eighty‐three percent of street‐tree species (*n* = 108, total) were used in a lower proportion than their availability by feeding birds, and nearly all were nonnative in origin. Our findings highlight the positive influence of street trees on urban avifauna. In particular, our results suggest that improved street‐tree management in lower‐income communities would likely positively benefit birds. Further, our study provides support for the high value of native street‐tree species and select nonnative species as important habitat for feeding birds.

## Introduction

Urbanization, the process of converting a natural ecosystem to one dominated by human development, is one of the most pervasive and dominant forms of land use globally (Foley et al. 2005, Grimm et al. 2015). Urbanization is a crucial process for providing living and working conditions for humans. However, the radical transformation of the landscape, coupled with the excessive requirements of cities for resources from outside their boundaries, has profound and negative impacts on ecosystems (Rees 1992, Collins et al. 2003). The pace of urbanization has greatly intensified worldwide over the past half century, with cities from around the world experiencing explosive densification and growth (Grimm et al. 2015). There is no slowdown in sight as countries and cities modernize and continue to provide amenities attractive for human habitation and relocation (Angel et al. 2011, Seto et al. 2012). Thus, the ecological footprints of urban areas will likely continue to grow, which poses critical challenges for biodiversity conservation (McKinney 2002, Lepczyk et al. 2017a).

The United States illustrates an example of a country that has undergone rapid urbanization, where, following the industrial revolution, cities have sprung up and sprawled, consuming much of the rural landscape (Angel et al. 2011, Grimm et al. 2015). One U.S. city in particular that exemplifies this pattern of growth is Los Angeles, California. Since the late 1800s, Los Angeles has grown from sparse homesteads and ranches situated across dusty agricultural fields to a major global metropolis (Stein et al. 2007). With the diversion of water from the Owens Valley in the early 20th century, Los Angeles boomed with people from across the United States and world moving to the California southland (Reisner 1987). A notable trend during the growth period in the early part of the 20th century and post‐WWII was the settlement of the region by residents from the American Midwest and Northeast (Pierson Doti and Schweikart 1989). Stately homes and neighborhoods with lawns and lush vegetation were developed, and city planners designed tree‐lined streets similar to what you would find in more mesic urban areas (Reisner 1987). Given the mild climate, the abundance of water from afar, and wealth, city planners created one of the most diverse and extensive urban forests in the world. We define “urban forest” as a collection of all trees within the boundaries of a metropolitan area (Nowak 2016). Estimates suggest there are well over a hundred tree species, with most being nonnative in geographic origin, planted throughout the entirety of Los Angeles (Clarke et al. 2013, Avolio et al. 2015).

**Fig. 1:**
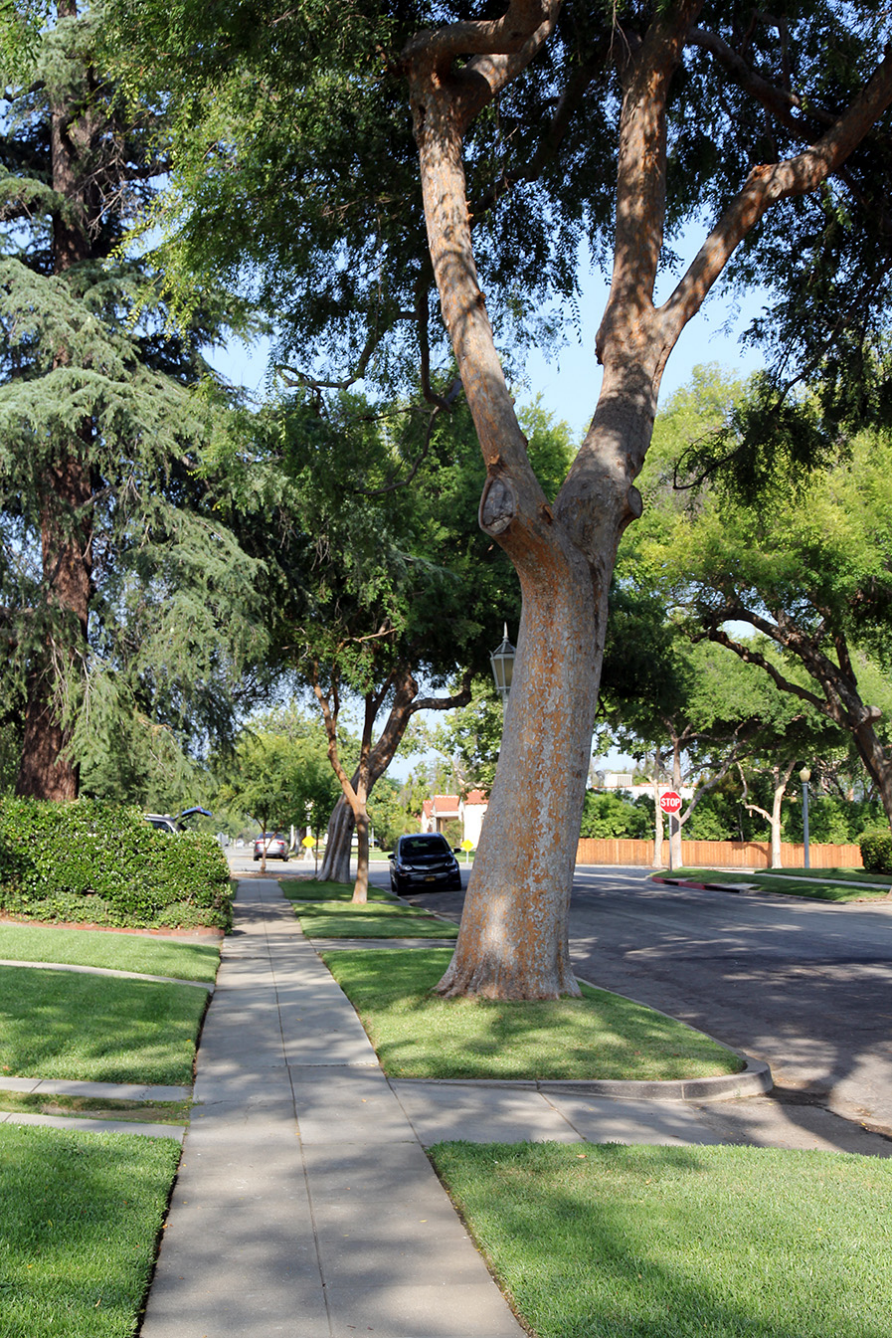
Street trees in a suburban neighborhood in Los Angeles County, California, USA (Photo credit, E. Wood).

One distinct component of urban forests throughout the world, including Los Angeles, are street trees (McPherson et al. 2016). Street trees are public resources and are therefore planted by municipalities in rights‐of‐way (e.g., sidewalk strips, Fig. 1; City Plants 2019). Street trees are planted for a variety of reasons and provide numerous functional services that benefit urban residents (McPherson et al. 2016). For example, street trees improve the aesthetical quality of cities (Southworth 2005), provide valuable environmental benefits (Livesley et al. 2016), and are positively associated with improved quality of life (Nowak et al., 2010). Further, street trees provide habitat for animals (Bhullar and Majer 2000, Shackleton 2016, Gray and van Heezik 2016) and thus likely provide a valuable role in urban biodiversity conservation (Nowak et al. 2010). Due to their importance, many cities have well‐developed street‐tree plans (City of Los Angeles 2004) and work to promote, maintain, and provide an inventory of trees within a city’s boundary (McPhearson et al. 2010, 2011).

Street trees are prevalent throughout cities in California, accounting for approximately 10–20% of the trees within the state’s urban forests (McPherson et al. 2015). Despite their commonness, the maintenance costs of street trees are likely high due to the excessive need for water to encourage growth in the arid environment (City Plants 2019). Further, while street trees are public resources, it is typically the responsibility of the property owner to maintain a tree adjacent to a residential unit (City Plants 2019). Because of the cost associated with maintaining street trees, lower‐income communities in some cities harbor a lower density of street trees and less urban forest cover than affluent communities (Landry and Chakraborty 2009, Kuruneri‐Chitepo and Shackleton 2011, Schroeter 2017). We define “street‐tree density” as the total number of street trees over a given area (Nowak et al. 2001), and “urban forest cover” as the area covered by the tree canopy throughout an urban ecosystem (Walton et al. 2008). One hypothesis put forth to explain the disparity in urban forest cover along a socioeconomic gradient is the “luxury‐effect hypothesis” (Leong et al. 2018), also termed the “inequity hypothesis” (Landry and Chakraborty 2009), which states that wealthy neighborhoods can withstand the financial costs of maintaining and caring for public and private trees while impoverished neighborhoods cannot. The luxury‐effect pattern is consistent across many cities in the world in explaining urban forest cover (Schwarz et al. 2015, Aronson et al. 2017, Avolio et al. 2018, Leong et al. 2018).

Further, there is additional support for the luxury effect extending to street trees (Brooks et al. 2016). Illustrating this, in Tampa Bay, Florida, and New York City, New York, lower‐income communities harbored less street‐tree cover than affluent areas (Landry and Chakraborty 2009, Schroeter 2017). In the Eastern Cape of South Africa, street‐tree diversity was higher in wealthy suburbs (Kuruneri‐Chitepo and Shackleton 2011). While it is clear that patterns in urban forest and street‐tree cover differ sharply across a socioeconomic gradient in many cities, it is unknown whether any apparent variability in street‐tree composition, density, and size influences urban bird communities.

Los Angeles is home to a high diversity and abundance of birds (Higgins et al. 2019), which consists of hundreds of migratory and non‐migratory species that utilize the urban ecosystem throughout the annual cycle (Garrett et al. 2012). One component of Los Angeles’ avian community that is prevalent during the winter months are migratory forest‐breeding birds (e.g., Yellow‐rumped Warbler, *Setophaga coronata*), which spend upward of six months of the annual cycle feeding on tree and shrub surfaces as they prepare for the spring migration and summer breeding season (Garrett et al. 2012). The other dominant component of the southern California avian community are non‐migratory birds, which are species that reside in natural habitats, such as chaparral, or urban environments throughout the year (Garrett et al. 2012, Higgins et al. 2019). While birds are seemingly ubiquitous throughout Los Angeles, their ecology in the urban ecosystem remains poorly understood, including their use of street trees. Providing wildlife habitat is a goal of many urban forest plans (Nowak and Dwyer 2000). However, there is no comprehensive assessment for the value of street trees to urban biodiversity in Los Angeles, or likely most cities around the world, which presents a critical gap in our understanding of conservation in urban ecosystems.

To understand the importance of street trees to wildlife, we designed a study where we measured and identified public street trees and documented foraging behavior of birds across two winters in residential communities situated across a socioeconomic gradient throughout Greater Los Angeles (hereafter LA). LA is an optimal place for studying the ecology of birds and street trees primarily because of the sheer extent and diversity of street trees within the urban forest as well as the stark differences in canopy cover throughout the metropolitan area. Further, birds are an optimal group for studying the importance of street trees to wildlife primarily because of their abundance and ability to reach nearly all areas of the urban ecosystem.

We had three objectives for our study. First, we documented patterns of street‐tree composition, diversity, density, and size, as well as feeding bird composition, diversity, and density across a socioeconomic gradient. We predicted that there would be distinct street‐tree communities across the socioeconomic gradient, with higher diversity and size of trees in more affluent areas, which is in line with the luxury‐effect hypothesis (Landry and Chakraborty 2009, Kuruneri‐Chitepo and Shackleton 2011, Brooks et al. 2016, Schroeter 2017). Further, we predicted that there would be distinct avian communities as well as more feeding birds in affluent areas, in part because of expected patterns of bird abundance in urban areas with higher vegetation cover (Blair 1996). Second, we quantified relationships between street‐tree diversity, density, and size and feeding bird density. We predicted that feeding birds would be positively related to greater street‐tree diversity, density, and size, primarily because of associations between birds and large and dense tree canopies in urban environments (DeGraaf and Wentworth 1986). Third, we evaluated whether there were patterns in foraging preferences of birds between native and nonnative street‐tree species. We predicted that birds would prefer native rather than nonnative trees, as native vegetation in urban environments provides abundant food resources for birds (Narango et al. 2017).

## Methods

### Study area

**Fig. 2:**
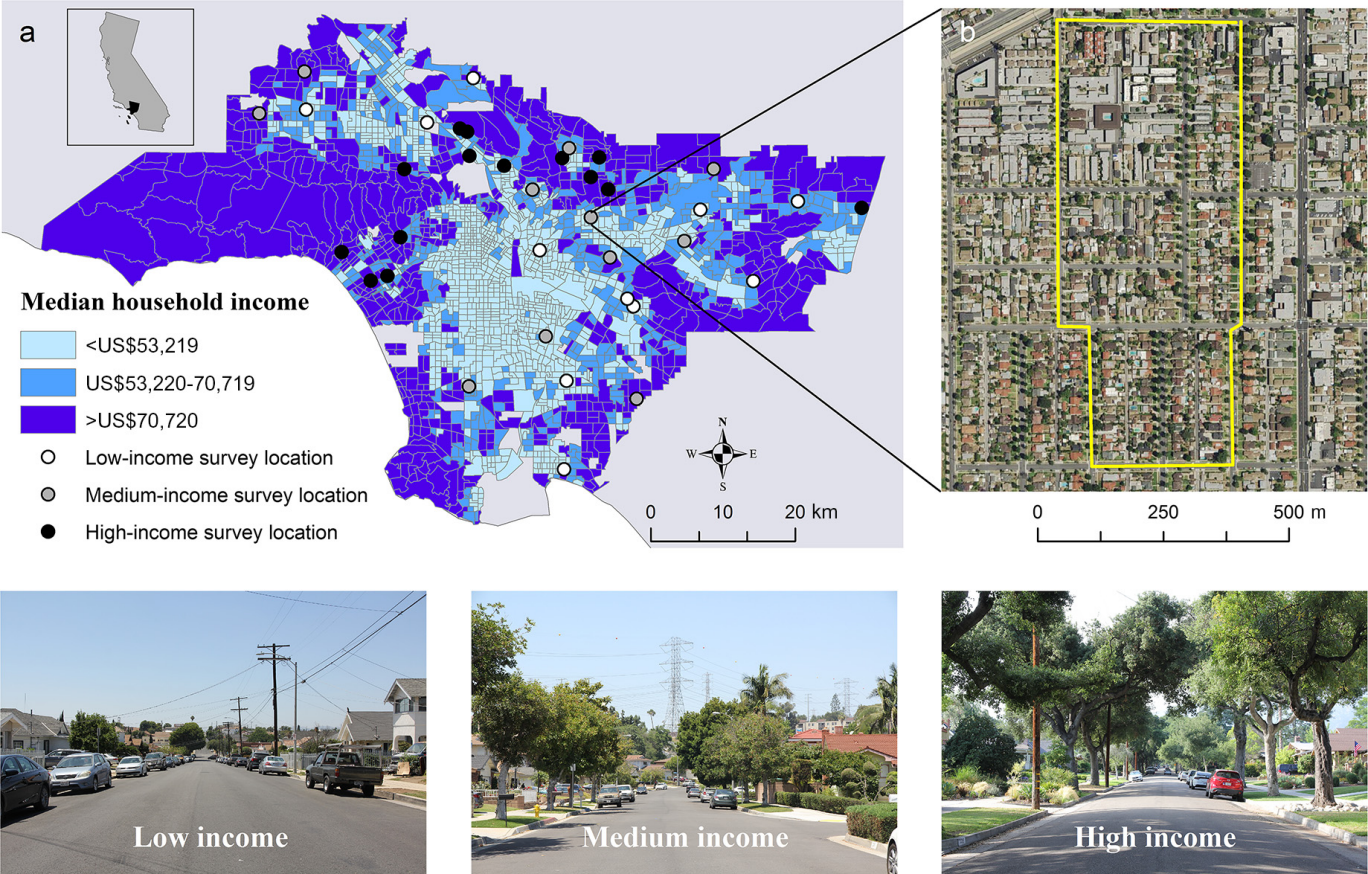
(a) Sampling design depicting 36 survey locations distributed across a socioeconomic gradient throughout the Los Angeles basin and surrounding valleys and mountains, Los Angeles County, California. (b) Inset map highlights a walking route (yellow line), where observers documented bird‐feeding behavior in street trees, twice during each of the 2016–2017 and 2017‐2018 winter seasons. Further, observers identified, recorded location, and measured diameter at breast height for all street trees throughout each route. Photos highlight typical differences in street trees from low‐, to medium‐, to high‐income areas of Greater Los Angeles (Photo credits, E. Wood).

We collected data on street‐tree diversity, density, size, and avian foraging behavior across a socioeconomic gradient in 36 residential communities throughout LA (Fig. 2a). The LA County metropolitan area is a sprawling mosaic of large and medium‐sized cities (e.g., Los Angeles, Long Beach, and Pasadena) and smaller municipalities (e.g., Culver City, Cerritos, and Montebello) that covers over 12,000 km^2^ and has a population of over 10,000,000 people (U.S. Census Bureau 2019; Fig. 2a). Mountainous protected areas ring the metropolis on the northern and eastern fringes, and the Pacific Ocean forms the southern and western boundary. The climate of the region is Mediterranean, characterized by cool, wet winters and hot, dry summers. The growing period typically follows the winter rains, and the native vegetation of the valley bottoms, which have been nearly fully developed, is a mosaic of wetland, grassland, shrubland, and woodland environments (Stein et al. 2007). Vegetation in the urbanized areas experiences variable growing conditions throughout the year, depending on irrigation patterns, planting practices, and geographic position in the city. For example, there are over 1,000 species of nonnative plants throughout LA (Avolio et al. 2019), and each likely has unique phenological patterns that may influence bird‐feeding behavior (Appendix S1). Patterns of precipitation and temperature are also highly variable throughout the region (yearly averages: 19°C/13°C high and low temperatures and 379 mm precipitation). In general, coastal communities have temperatures and precipitation patterns that are more moderate, whereas valley and mountain areas experience more extreme temperature ranges and periodic heavy precipitation that occasionally cause flooding in valleys.

The settlement history of LA created one of the most diverse and multicultural metropolises in the world (Pierson Doti and Schweikart 1989, Evanosky and Kos 2014). In addition to the multiculturalism of LA, the city contains a great range of wealth distribution (Fig. 2). Municipalities such as Beverly Hills and San Marino typify extreme opulence, whereas areas such as downtown LA’s skid row and communities in southcentral LA experience poverty, based on the U.S. Census poverty thresholds for a family of four in 2015 (<US$24,257, U.S. Census Bureau poverty thresholds, Fig. 2). The patterns of tree cover throughout LA reflect patterns of the income distribution, where lower‐income communities have far less "tree" cover than affluent ones (Avolio et al. 2015, Fig. 2). The spatial distribution of wealth follows a pattern where affluent communities tend to be located in the foothills of mountainous protected areas and open spaces, the immediate coastal zones, and the southeastern border with Orange County (Fig. 2a). In contrast, lower‐income communities are located surrounding downtown LA, East LA, southcentral LA, and central portions of the San Fernando Valley (Fig. 2a).

To address our study objectives, we established a survey design set in residential communities throughout LA. To identify residential communities along a socioeconomic gradient of survey interest, we used U.S. census tract data, combined with published records of median household income (Los Angeles Times 2015). To determine low‐, medium‐, and high‐income census tracts, we gathered median household income values, tabulated by the 2010 census, for 265 neighborhoods that were located within our study boundaries of Los Angeles County (Los Angeles Times 2015; Fig. 2a). The median household income based on the 2010 U.S. Census tract data was US$62,932, which was comparable to the U.S. Department of Housing and Urban Development (2015) median family income calculations for 2015 in Los Angeles County (US$63,000, data available online).^4^
https://www.huduser.gov/portal/home.html
 From the 2010 U.S. Census tract data, we determined the lower 33% as “low” (<US$53,219), the middle 33% as “medium” (US$53,220 to US$70,719), and the upper 33% as “high” (>US$70,720). We initially considered 2163 census tracts for inclusion in our sampling design. One thousand and eighty one census tracts were in low‐income communities (49.98% of the total), 470 in medium‐income communities (21.73%), and 612 in high‐income communities (28.29%, Fig. 2). Low‐income census blocks covered approximately 25% of the available area for study, whereas medium‐ and high‐income communities covered 19% and 56% of the available area for study, respectively (Fig. 2).

After categorizing census tracts based on socioeconomic levels, we used a spatially balanced random‐tessellation approach (Stevens and Olsen 2004) in conjunction with ArcGIS software (ESRI 2016) to identify 60 census tracts with 20 in each of low‐, medium‐, and high‐income brackets. We then used Google Earth combined with Google Street View (Google 2016) to identify residential areas within selected census tracts with streets bordered by sidewalks that separated private front yards from street trees (Fig. 1). Some sections of LA, especially more affluent regions, lacked sidewalks, and we excluded those from our survey for safety precautions and because of the ambiguity over whether trees were considered public (i.e., a street tree) or private (i.e., a tree in a yard) due to no noticeable right‐of‐way separating private yards from streets. Further, we avoided streets with no discernable zone for street trees, areas where surveys were challenging due to pedestrian and vehicle traffic (e.g., major thoroughfare roads, freeway on/off ramps, commercial zones, and industrial areas), public spaces that were not residential (e.g., city parks), and sections of the city where safety was a concern. After further scrutiny of the 60 identified census tracts, we refined our initial selection based on our sampling requirements, leaving us with 36 survey locations, with 12 located in each of low‐, medium‐, and high‐income census tracts. Within each of the 36 survey locations, we plotted walking routes using Google Earth software (Google 2016) that were approximately two and a half km in length (average, 2.49 km), which we used for all street‐tree sampling and bird‐foraging behavioral work (Fig. 2b). The boundary surrounding the extent of our survey locations encompassed an area of approximately 4,395 km^2^ and included the foothills of major mountain ranges, the main valleys of LA, including the LA Basin, the San Fernando Valley, and the San Gabriel Valley, and the western portions of the Inland Empire (Fig. 2). The distance between the centroids of survey locations ranged from 1.08 to 12.67 km, with an average length of 5.10 km (Fig. 2). Our sampling design yielded independent data, which was necessary for statistical analyses (Appendix S1, Fig. S1).

Due to the rapidly shifting housing market in LA and our selection of routes that contained street trees and other amenities such as sidewalks that are likely associated with increased housing value, it was apparent that we misclassified some survey locations based on the 2010 census data. Thus, before our analysis, we further refined our socioeconomic classifications based on estimated housing values from the Redfin real estate website (Redfin 2018). During the fall of 2018, we gathered estimated real estate values for all single‐unit homes, as well as values for single units within multi‐unit residences (e.g., apartment complex) with frontage property on walking routes (*n* = 6,292) and calculated the range (US$59,000–US$26,100,000), the median (US$677,000), and the lower (<US$593,000 USD) and upper‐third (>US$809,000 USD) percentiles. Further, we gathered data on the parcel size and the number of all single‐unit residences on walking routes. We calculated the range (parcel size, 155.61–5053.83 m^2^; single‐unit homes per 1 km of walking route, 36–130), the median (parcel size, 668.81 m^2^; single‐unit homes per 1 km of walking route, 59), and the lower (parcel size <609.91; single‐unit homes per 1 km of walking route, <51) and upper‐third (parcel size >703.36; single‐unit homes per 1 km of walking route, >65) percentiles. From the updated real estate values, we shifted one low‐income neighborhood to medium income, and two medium‐income neighborhoods to high income, leaving us with 11 survey locations in low‐, 11 in medium‐, and 14 in high‐income residential areas (Fig. 2).

### Public street‐tree measurements

We measured diameter‐at‐breast‐height (DBH) and recorded the tree species for each street tree along a walking route. To quantify street‐tree species availability as foraging substrates for birds, we calculated density, dominance, and the importance value of each tree species (Holmes and Robinson 1981, Gabbe et al. 2002, Wood et al. 2012). DBH is a strong predictor of tree crown diameter and height in both forest (Gering and May 1995) and street‐tree populations (Peper et al. 2001), and thus, we assumed is a surrogate for quantifying the availability of foraging substrate for arboreal feeding birds in our urban study system. Density represents the total number of a given tree species over a defined area, whereas dominance is a measure of the area covered by a street‐tree species. To calculate dominance, we converted DBH values of a measured tree into a basal area (Gabbe et al. 2002, Wood et al. 2012). We standardized the total counts of trees and basal area to 1‐km of walking route, which enabled us to calculate total tree density and total basal area in each survey location. We used the standardized total tree density and total tree basal area measurements of each survey location as independent variables in our objective one and two analyses. To calculate importance values for each tree species across all survey locations, we calculated the density and basal area for each street‐tree species, computed the relative values of both, and summed those to obtain importance values. We then divided the summed importance value by two to express the importance values as relative values (Gabbe et al. 2002, Wood et al. 2012). We used the relative importance values of street trees in our objective three statistical analyses. We did not include frequency in our calculation of street‐tree importance values as our survey was not based on plotless sampling within forest stands, which is necessary for calculating the frequency metric (Wood et al. 2012). Further, omitting frequency and instead focusing on density and size (dominance) of street trees, two variables that we predicted would influence feeding bird behavior (DeGraaf and Wentworth 1986), is an approach that has been employed by previous investigations of importance values of street‐tree populations in urban systems (McPherson and Rowntree 1989).

### Avian foraging observations

To characterize the foraging behavior of birds, we surveyed all street trees along walking routes for feeding birds, twice per winter, from October to March 2016–2017 and 2017–2018. We focused our surveys during the winter months to observe the diverse and abundant wintering migratory bird community (hereafter migratory birds). We conducted foraging observations 30 minutes following sunrise and ended within 4 h post‐sunrise. Wintering birds tend to flock and move in search of food during the non‐breeding period (Greenberg 2000). Therefore, we waited at least three weeks between visits within a season to allow for any possible turnover of birds that may have immigrated to or emigrated from a survey location to limit possible double counting of individual birds during repeat visits. Our protocol called for two observers to complete surveys, with one observer walking along one sidewalk on a street, and the other on the adjacent sidewalk, moving in concert throughout the survey. S. Esaian led all field surveys and was accompanied by E. Wood or trained student observers.

To quantify migratory bird‐foraging behavior on public street trees, we selected five, primarily arboreal feeding, migratory species that are common during the winter months in the LA urban forest. These included the Ruby‐crowned Kinglet (*Regulus calendula*), the Orange‐crowned Warbler (*Oreothlypis celata*), the Yellow‐rumped Warbler (*Setophaga coronata*), the Black‐throated Gray Warbler (*Setophaga nigrescens*), and the Townsend’s Warbler (*Setophaga townsendi*) (Appendix S1: Table S1). We selected these species because they represent a segment of the population of terrestrial Nearctic‐Neotropical migratory birds that spend the winter in southern California, they breed in more northern forested ecosystems during the summer, and they frequently forage on tree surfaces and thus were commonly encountered during our surveys (Garrett et al. 2012). Additionally, their populations are generally in decline, highlighting the importance of understanding the role of street trees in urban forests for the conservation of migratory birds (Sauer et al. 2017).

When we detected one of the five migratory bird species actively feeding on the surface of a street tree, we recorded foraging behaviors for up to three minutes (average time = 47 s). Each observation included documenting the tree species along with the bird’s foraging behavior, including all search efforts (walk and shuffles, hops, and flights) and attacks (a glean on the surface of leaves, bark, flowers, or seeds, or aerial maneuver; Remsen and Robinson 1990, Wood et al. 2012). To prevent pseudo‐replication of foraging observations, we recorded feeding behavior only of individuals of the same species >100 m from where we ceased a previous observation unless there were apparent differences between male and female individuals. Our methodology to avoid pseudo‐replication may have masked our ability to detect more feeding birds in areas with higher tree density. Nevertheless, we decided on our approach to prevent the double counting of bird observations as we walked along routes. We frequently observed individual migratory birds foraging in multiple street trees during observations. We recorded each new tree species in which we documented a bird feeding. A handful of tree species provided challenging conditions for observing foraging birds due to their dense canopy (e.g., the Canary Island pine [*Pinus canariensis*]). If a tree canopy was overly dense, and we detected a study bird, we observed the individual until we recorded a feeding observation, which was a documentation of “use”. We then ceased the observation. If we did not detect a bird feeding after three minutes in challenging‐to‐observe trees, we resumed our survey of other trees along the walking route. The latter scenario occurred for < 1% of our total observations.

To understand patterns of street‐tree use by a segment of the bird population that is prevalent in LA throughout the annual cycle, we focused on five species that regularly forage in trees. These included the Allen's Hummingbird (*Selasphorus sasin*), the Anna's Hummingbird (*Calypte anna*), the Bushtit (*Psaltriparus minimus*), the Lesser Goldfinch (*Spinus psaltria*), and the House Finch (*Haemorhous mexicanus*) (Appendix S1: Table S2). Segments of Allen’s and Anna’s Hummingbird populations migrate northward during the breeding season (Garrett et al. 2012, Greig et al. 2017). However, these two species are common in LA throughout the year (Allen et al. 2016, Clark 2017). The other three species are non‐migratory. Therefore, we refer to this group as “year‐round” birds.

In addition to feeding on the surfaces of trees, we selected these five year‐round species as each has preferences for unique food resources that were present throughout the survey period. For example, the hummingbirds are often attracted to exuberant flowering, Bushtits to leaf surfaces, and the finch species to seeds (Allen et al. 2016). Therefore, studying these five species enabled us to understand how birds with different feeding behaviors and food needs interact with the high diversity of street trees and shifting phenophases throughout the winter season (Appendix S1). When we detected a year‐round species feeding on a street tree, we again recorded use and the specific substrate in which we observed a feeding attempt (e.g., leaf, bark, flower, seed, or aerial maneuver). We did not collect detailed foraging behavior on year‐round birds, because their foraging behavior was often stationary (e.g., a House Finch feeding on a seed capsule of an American Sweetgum, *Liquidambar styraciflua*). Similar to our observations of migratory birds, to prevent double counting of year‐round birds, we collected foraging observations only of individuals of the same species >100 m from the last observation unless it was clear they were different individuals (e.g., visual differences between male and female House Finches).

We expected that additional factors other than the street tree in which we observed a feeding bird might influence foraging behavior. For example, affluent areas often have decadent yards, full of vegetation, which may attract feeding birds (Lerman and Warren 2011, Clarke et al. 2013). Additionally, some residential communities are near protected areas or open spaces and thus could provide easier access for birds that prefer more natural environments (Donnelly and Marzluff 2004). In a parallel study, we counted birds throughout LA and documented whether we observed birds using either public features, which included street trees or utility lines, or vegetation in private yards (E. M. Wood and S. Esaian,* unpublished data*). Further, in that study, we recorded distance from survey locations (centroid of survey routes) to the nearest federal protected area or open space. We observed 50.1% of detected birds (*n* = 3,691) in street trees (either feeding, vocalizing, or resting) or utility lines (primarily species of *Columbidae*), whereas the other 49.9% of observations (*n* = 3,679) were in private yards, flying over count locations, or in areas where we could not determine their usage (e.g., singing from an adjacent street). While we commonly observed birds maneuvering back and forth between vegetation in yards and street trees, it was equally as common to observe birds moving from street‐tree to street tree as they fed. In low‐income communities, nearly all feeding birds that we detected were foraging in street trees, as there is little yard vegetation (Fig. 2). Last, we found no correlations between the density of feeding birds and street‐tree density and size with distance to protected area or open space (Spearman’s rho, ρ = 0.01–0.27, *P* = 0.10–0.94). Therefore, we assumed that our study design and survey methodology likely characterized the foraging behavior of birds based on their ecology with a given street‐tree species as opposed to external factors that may have influenced their feeding patterns.

### Statistical analysis

To address our first objective of documenting patterns of street‐tree composition, diversity, density, and size, as well as feeding bird composition, diversity, and density across the socioeconomic gradient, we completed two separate analyses for both trees and birds, respectively. First, to identify the degree of dissimilarity in street‐tree communities across the socioeconomic gradient, we conducted a one‐way analysis of similarities test (ANOSIM; Oksanen 2019), using the Bray‐Curtis dissimilarity of the square‐root transform of counts of street trees, grouped by socioeconomic classification. The ANOSIM analysis is a nonparametric test that uses Monte Carlo randomization of observed data to assess whether ranked dissimilarities within socioeconomic groups were more similar than among groups (Oksanen 2019). We used 999 Monte Carlo permutations to generate the random test statistic, *R*, which ranges from −1 to 1. An *R* value near zero indicates that the street‐tree community does not differ among socioeconomic groups, whereas *R* values further from zero indicate increasing dissimilarity. As we made three comparisons among the three socioeconomic groups, we used a Bonferroni adjustment to the alpha value of 0.05/3 = 0.017 to assess significance. We computed the ANOSIM analysis using the “vegan” package in R (Oksanen 2019).

In a secondary analysis, we explored differences in street‐tree diversity, which we expressed as species richness and the Shannon diversity, density, and basal area across the socioeconomic gradient. As our walking routes within survey locations were all slightly different distances, we standardized our tree species richness data to one km of walking route, which was similar to our adjustments of tree density and basal area. We used either a one‐way analysis of variance (ANOVA) or a Kruskal‐Wallis test, depending on whether assumptions for parametric linear models were satisfied, with the socioeconomic group as the fixed, categorical factor. When ANOVA or Kruskal‐Wallis tests were significant, we computed a multiple comparisons routine using either a parametric Tukey’s HSD test or a nonparametric procedure, based on relative contrast effects (nparcomp package in R; Konietschke 2011). We evaluated pairwise comparisons among groups using a Bonferroni adjusted alpha value (0.05/3 = 0.017).

To quantify differences in feeding bird composition and foraging observations across the socioeconomic gradient, we again computed an ANOSIM analysis, and an ANOVA test, following a similar approach to the street‐tree analysis. To compute our bird‐foraging response variable, we determined an *n* = 1 as a unique feeding attempt of a bird on a tree substrate. If we detected a single bird feeding on multiple trees, we used only the foraging behavior and substrate of that bird on the first tree on which we observed it. For year‐round birds, some species aggregated into large flocks while moving and feeding (e.g., Bushtits and House Finches). If we detected a large flock feeding on a similar tree species, we recorded each flock as one observation to avoid overinflating the ecological importance of a given tree on the movement and feeding patterns of a group of birds. If we detected a mixed‐species flock feeding, we recorded an *n* = 1 for each year‐round bird species represented within the flock. To determine whether we were underestimating effect sizes by our treatment of flock size, we calculated a Spearman’s rho (ρ) correlation between our reduced measure of flocks with tallies of all individuals within flocks. We found both metrics to be highly correlated (Spearman’s ρ = 0.81, *P* < 0.01). This analysis suggests our approach yielded data and results comparable to full flock tallies (Appendix S1). To quantify the number of feeding birds at each survey location, we summed the feeding observations for either the migratory or year‐round birds at each survey location across the four visits. Similar to our street‐tree richness, density, and size variables, we standardized our bird observation feeding data to one km of a walking route. We thus refer to our feeding observations as “feeding‐bird density” measures.

To address our second objective of quantifying relationships between street‐tree diversity, density, and size with feeding‐bird density, we fit a series of nine single‐variable generalized linear models (Table 2). We fit three model sets, in which each set consisted of one of three dependent variables, eight independent variables, and the intercept‐only model. The dependent variables were (1) the number of observations of feeding migratory birds, standardized per 1 km of a walking route (migratory bird density); (2) the number of observations of feeding year‐round birds, standardized per 1 km of a walking route (year‐round bird density); and (3) the total number of observed feeding birds, standardized per one km of a walking route (total bird density). In general, we did not notice substantial differences in bird observations between years (Appendix S1: Tables S1, S2). Therefore, we combined avian observation data across the two winter seasons to understand relationships between feeding‐bird density and street‐tree attributes based on the four visits to each survey location.

We selected eight independent variables that captured both street‐tree diversity (species richness and Shannon diversity), as well as the structural attributes of street‐tree density and size that may influence bird behavior (DeGraaf and Wentworth 1986). Further, in addition to analyzing the density and size of all street trees, we grouped street trees, whether they were native or nonnative, to understand whether the geographic origin of a tree species influenced feeding‐bird density (Appendix S1: Table S3). We considered trees native if they naturally occur in the LA basin, adjacent valleys, and surrounding foothills and nonnative if they naturally occur elsewhere, whether in California outside of the south coast portion of the state, in the United States outside California, or in a different country (Appendix S1: Tables S3). To determine the distribution of trees, we used range maps from the CalFlora database (CalFlora 2019). To assess the strength and directionality of the relationship of each independent variable with a dependent variable, we also fitted the intercept‐only model to compare with the dependent variable mean of a model set.

Because our dependent data were density estimates derived from discrete observation variables, we approached our model fitting using Poisson generalized linear models (Zuur et al. 2011). When viewing initial scatterplots, we noticed the variance did not appear to equal the mean, an assumption of Poisson generalized linear models (Zuur et al. 2011). Instead, the variance typically appeared to broaden, depending on the level of the fitted relationship. Thus, to ensure an accurate characterization of the variance of the fitted relationship, we considered either a Poisson distribution or a negative‐binomial distribution (both fit using a log‐link function; Zuur et al. 2011). To determine whether to use a Poisson or a negative‐binomial distribution for each model, we first fitted a Poisson generalized linear model for each relationship. We then assessed the fit of each model by calculating the Pearson χ^2^ statistic and evaluated the level of overdispersion by calculating the ratio of the residual deviance to the residual degrees of freedom (Zuur et al. 2011). In all cases, fitting a model using the Poisson generalized linear modeling approach yielded a substantial lack of fit, with clear evidence for overdispersion. Thus, we proceeded to fit models using a negative binomial distribution to account for the overdispersion evident in our data (Zuur et al. 2011). After fitting a negative binomial model, we again calculated the Pearson χ^2^ statistic and checked for overdispersion (Zuur et al. 2011). In all cases, negative binomial models were an adequate fit to the data, and thus, we used this distribution for all fitted models. We computed all generalized linear models using the MASS package in R (Venables and Ripley 2002).

Many relationships displayed hump shapes. In these cases, we fitted the generalized linear models with a quadratic term to account for the hump‐shaped relationship. There were no further intricate shapes (e.g., third‐ or fourth‐order polynomial) apparent between variables. To evaluate the fit of the models within each set relative to one another, we used Akaike’s Information Criterion (AIC) and a model‐selection framework.

To address our third objective of evaluating whether there were patterns in foraging preferences of birds among both native and nonnative street trees, we completed two analyses.

First, to determine whether birds fed on street trees species in differing proportions than they were available throughout the cityscape, we computed a χ^2 ^goodness‐of‐fit test. To calculate the analysis, we compared observed feeding vs. expected feeding frequencies for migratory, year‐round, and total‐feeding observations for seven of the 10 study bird species for which we had sufficient observations (*n* ≥30 feeding observations). We used 21 street‐tree species, all of which had an importance value percentage >1.5% as we assumed birds rarely used uncommon street‐tree species. 

Second, to estimate the selectivity of migratory birds for street‐tree species, we calculated preference and aversion values (Holmes and Robinson 1981, Wood et al. 2012). Preference and aversion values are the difference between relative importance values of each street‐tree species with that of observed feeding proportions of birds (Gabbe et al. 2002, Wood et al. 2012). Preference and aversion values do not determine resource selection, which requires equal abundance of available resources, but they may represent a bird’s preference (positive values) and aversion (negative values) of foraging substrates. We calculated preference and aversion values for the same bird groups and species as the χ^2^ goodness‐of‐fit analysis. We used the R statistical software for all analyses and graph creation (R Core Team 2017).

## Results

Throughout the two winter field seasons, we surveyed approximately 90 km of street on four occasions, over which we identified, measured, and recorded the position of 7,637 street trees of 85 species (Appendix S1: Table S3). Five tree species were native, and the remaining 80 were nonnative, accounting for 5.46% and 80.51% of the total street‐tree importance, respectively. Further, in addition to the 85 tree species, we encountered 23 tree families, which were composed of challenging to identify street trees belonging to the same family (e.g., *Fraxinus* spp., Appendix S1: Table S3). These families were most likely comprised of nonnative trees and accounted for 11.50% of the total street‐tree importance. Last, we encountered 257 individual nonnative trees that we were unable to identify to species or family. The unknown nonnative group made up the remaining 2.53% of street‐tree importance (Appendix S1: Table S3).

Of the native tree species, the coast live oak (*Quercus agrifolia*) and the California sycamore (*Platanus racemosa*) were the only commonly encountered tree species throughout LA (Appendix S1: Table S3). We measured 236 coast live oaks and 79 California sycamore trees, and the average DBH of each species was 76.01 cm and 94.85 cm, respectively (Appendix S1: Table S3). The most commonly encountered street trees of our study were nonnative, with the southern magnolia (*Magnolia grandiflora*), common crape myrtle (*Lagerstroemia indica*), American sweetgum, camphor tree (*Cinnamomum camphora*), and Chinese elm (*Ulmus parvifolia*) being the most abundant (*n* = 700, 592, 546, 530, and 499 individuals, respectively, Appendix S1: Table S3). The street‐tree species covering the greatest area were the camphor tree (*n* = 404.18 m^2^ basal area/km), Italian stone pine (*n* = 384.74 m^2^/km), and Chinese elm (*n* = 330.67 m^2^/km, Appendix S1: Table S3).

We recorded 938 observations of feeding birds, totaling over 10 h of observation time. We documented 587 observations of migratory birds and 351 of year‐round birds (Appendix S1: Tables S1 and S2). The most commonly encountered migratory bird was the Yellow‐rumped Warbler (*n* = 348 feeding observations), followed by the Ruby‐crowned Kinglet (*n* = 136 observations), the Townsend’s Warbler (*n* = 69 observations), the Orange‐crowned Warbler (*n* = 23 observations) and the Black‐throated Gray Warbler (*n* = 10 observations, Appendix S1: Table S1). The most commonly encountered year‐round bird was the Bushtit (*n* = 141), followed by the House Finch (*n* = 96), the Lesser Goldfinch (*n* = 61), the Anna’s Hummingbird (*n* = 30), and the Allen’s Hummingbird (*n* = 23, Appendix S1: Table S2). Overall, there was little variability between field seasons in observations of migratory and year‐round birds (Appendix S1: Tables S1, S2). The only notable differences were for Yellow‐rumped Warblers (*n* = 203, 145), Townsend’s Warblers (*n* = 23, 46), Allen’s Hummingbirds (*n* = 15, 8), and House Finches (*n* = 64, 32) (Appendix S1: Tables S1, S2).

### Objective #1:street‐tree and bird composition, diversity, and density

Street‐tree and feeding bird composition were significantly dissimilar among low‐, medium‐, and high‐income areas (street‐tree ANOSIM *R* = 0.13, *P* < 0.01; feeding bird ANOSIM *R* = 0.28, *P* < 0.01). For both street trees and birds, low‐ and high‐income areas were most dissimilar (street‐tree ANOSIM *R* = 0.20, *P* < 0.01; feeding bird ANOSIM *R* = 0.55, *P* < 0.01), followed by medium‐ and high‐income areas (street‐tree ANOSIM *R* = 0.14, *P* = 0.02; feeding bird ANOSIM *R* = 0.24, *P* < 0.01), and low‐ and medium‐income areas, which were not significantly dissimilar (street‐tree ANOSIM *R* = 0.02, *P* = 0.32; feeding bird ANOSIM *R* = 0.01, *P* = 0.33).

**Fig. 3:**
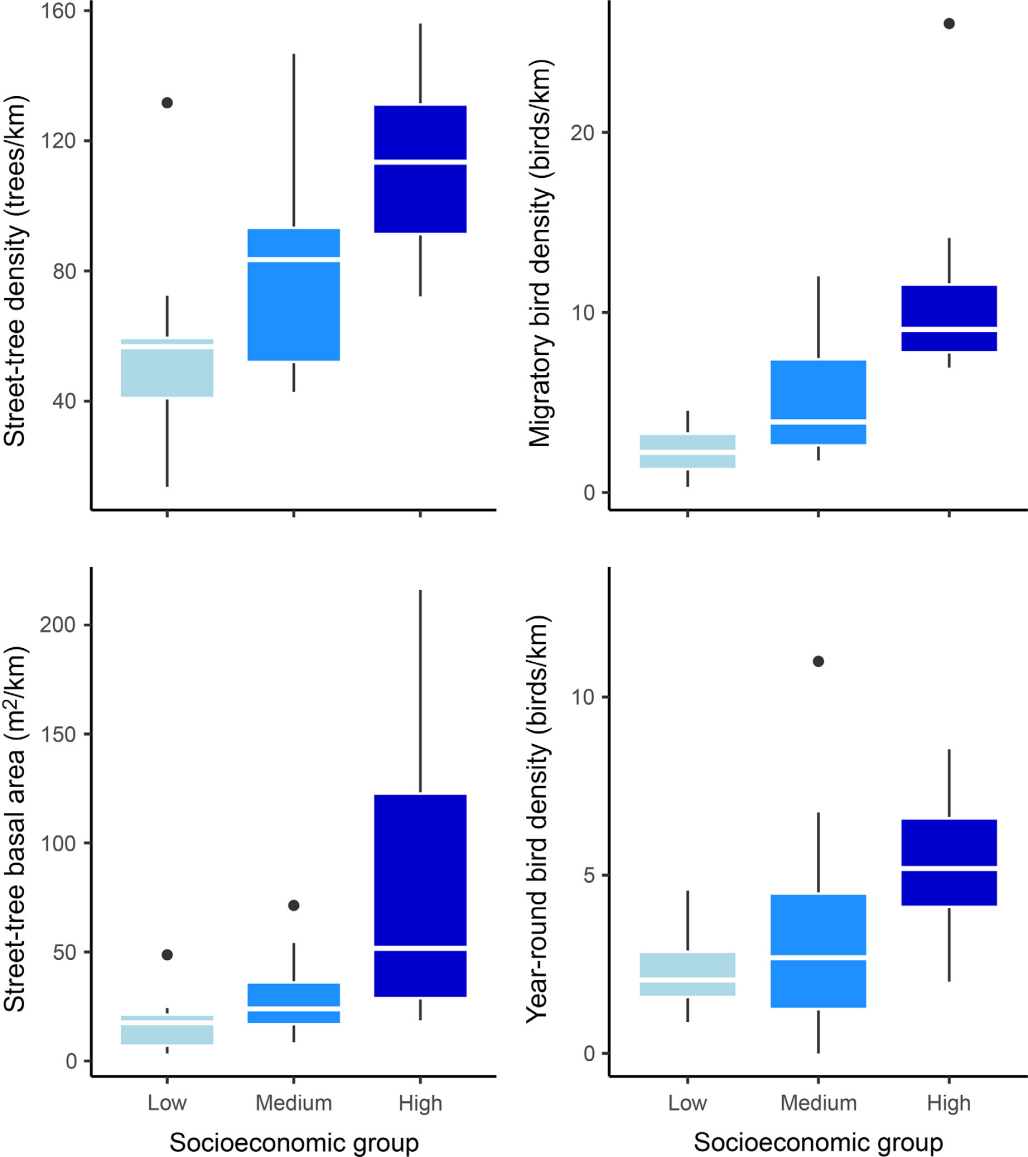
Box‐plot summaries of street‐tree density (number of street trees per 1 km of survey route), total street‐tree basal area (m^2^) per km, and migratory and year‐round feeding bird density within 36 residential communities situated across a socioeconomic gradient of low (<US$53,219, median household income), medium (US$53,220–US$70,719), and high‐income residential communities (>US$70,720) throughout Greater Los Angeles. In all cases, high‐income residential communities harbored significantly greater tree density, tree basal area, and density of migratory and year‐round feeding birds than medium and low‐income residential communities based on a one‐way ANOVA or Kruskal‐Wallis analysis followed by a multiple comparisons analysis. The boxplot figures display the median values, the first and third quartile, and the minimum and maximum values, while circles denote outliers.

Migratory and year‐round birds were five and two times denser, respectively, in high‐ compared with low‐income survey areas, and approximately two times as dense in high‐ compared with medium‐income survey areas, and medium‐ compared with low‐income areas (*F*_2,33_ = 15.63 and 5.18, *P* ≤ 0.01, Table 1, Fig. 3). Tree species richness was similar across the socioeconomic gradient (*F*_2,33_ = 0.75, *P* = 0.48, Table 1). However, lower‐income communities had a higher Shannon diversity than medium and high‐income regions of the city (*F*_2,33_ = 3.20, *P* = 0.05, Table 1). Street trees were twice as dense and nearly five times greater in size in high‐income areas compared with low‐income areas (Kruskal‐Wallis χ^2^ = 7.31 and 13.54, *P* < 0.03, Table 1, Fig. 3). High‐income areas were also significantly different in tree density and size compared with medium‐income areas, while medium‐ and low‐income areas were similar (Table 1). Nonnative trees followed a similar pattern (Kruskal‐Wallis χ^2^ = 13.21 & 11.99, *P* < 0.01, Table 1). Due to low sample sizes, we did not detect significant differences in native tree density and size across the socioeconomic gradient (Table 1). However, native trees in high‐income areas were 14 times as dense and covered nearly ten times the area compared with low‐income residential areas.

**Table 1:** Summaries of feeding bird density, street‐tree diversity, and street‐tree density and size variables, standardized per 1 km of survey route, across a socioeconomic gradient of low‐ (<US$53,219, median household income), medium‐ (US$53,220–US$70,719), and high‐income residential communities (>US$70,720) throughout the Los Angeles (California, USA) metropolitan area.

Parameter	Low	Medium	High
Feeding bird density			
Migratory birds	2.29^A^ ± 0.22	5.31^A^ ± 0.62	10.66^B^ ± 0.83
Year‐round birds	2.37^A^ ± 0.19	3.53^A^ ± 0.53	5.17^B^ ± 0.30
All feeding birds	4.66^A^ ± 0.31	8.83^A^ ± 1.06	15.83^B^ ± 0.97
Street‐tree diversity			
Street‐tree species richness	9.06 ± 0.52	9.08 ± 0.65	7.68 ± 0.50
Street‐tree Shannon diversity†	2.46^A^ ± 0.09	2.25^A^ ± 0.09	1.87^B^ ± 0.11
Street‐tree density and size			
Total street‐tree *n*	54.10^A^ ± 5.25	80.47^A^ ± 5.49	112.84^B^ ± 4.12
Native street‐tree *n*	0.54 ± 0.13	1.07 ± 0.30	7.85 ± 1.67
Nonnative street‐tree *n*	53.56^A^ ± 4.05	79.40^AB^ ± 5.19	104.98^B^ ± 5.28
Total street‐tree basal area (m^2^)	16.79^A^ ± 2.15	29.16^A^ ± 3.23	79.67^B^ ± 10.80
Native street‐tree basal area (m^2^)	0.70 ± 0.30	0.35 ± 0.11	6.42 ± 1.92
Nonnative street‐tree basal area (m^2^)	16.09^A^ ± 2.68	28.81^A^ ± 4.80	73.25^B^ ± 10.47

Notes

Variables with the same superscript letter do not differ significantly among socioeconomic groups based on a one‐way ANOVA with Tukey HSD test or Kruskal‐Wallis test with nonparametric multiple comparisons procedure, with Bonferroni adjusted *P* value: 0.05/3 = 0.02. Values are mean ± SE.

† Not standardized to 1 km of walking route.

### Objective #2: relationships between street trees and feeding bird density

**Fig. 4:**
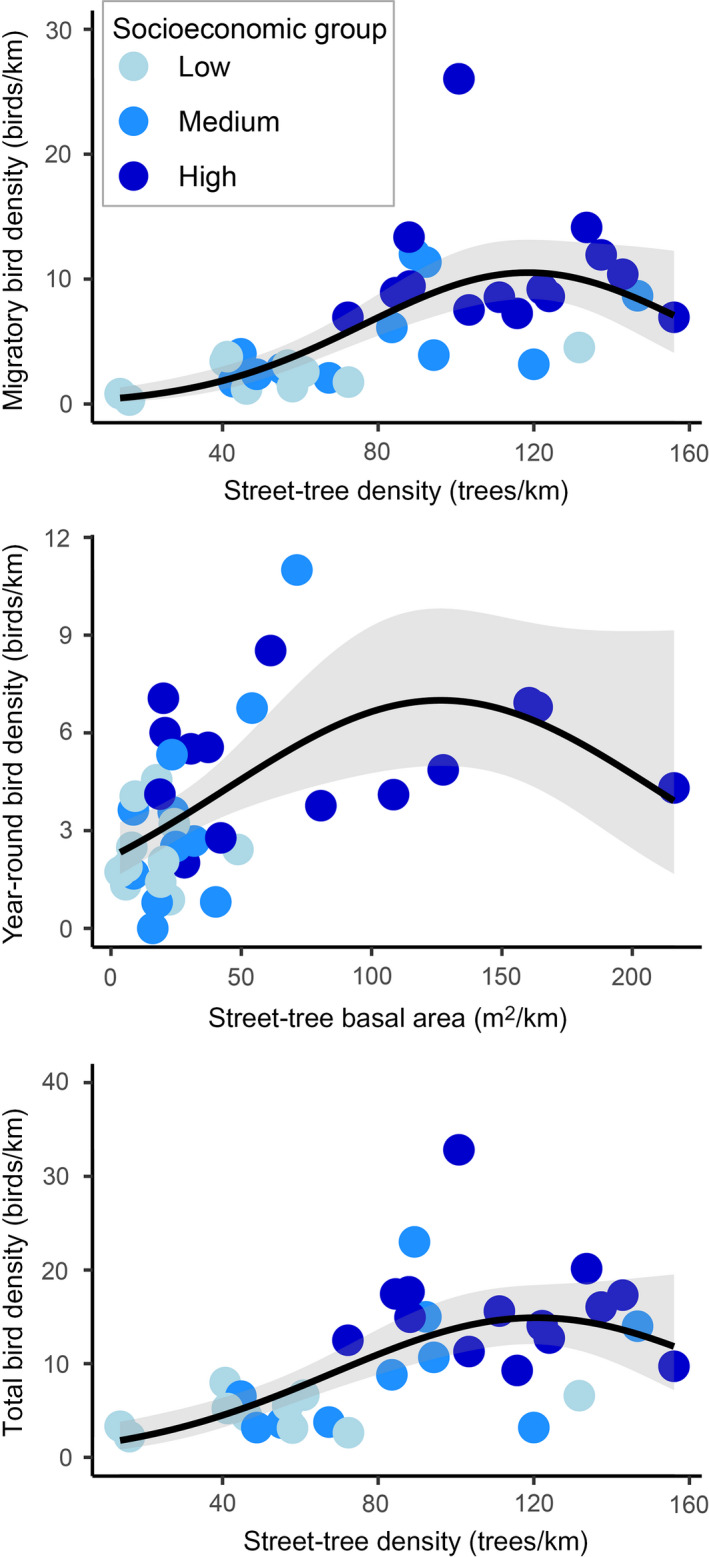
Scatterplots depicting the relationships between density of feeding migratory, year‐round, and total birds (migratory and year‐round feeding birds combined) with street‐tree density and street‐tree size. We derived the fitted smoothed line and estimated prediction intervals from a generalized linear model analysis using a negative binomial error distribution. The color scheme represents survey areas located in 36 residential communities situated across a socioeconomic gradient of low (<US$53,219, median household income), medium (US$53,220–US$70,719), and high‐income residential communities (>US$70,720) throughout Greater Los Angeles.

The top‐fitting independent variable describing migratory bird density was total street‐tree density, which had a ∆AIC value of 2.66 less than the second‐best model. The ∆AIC value for the intercept‐only model was 27.79, suggesting strong support that total street‐tree density best explained migratory bird‐feeding density throughout our LA study area (Table 2, Fig. 4). The overall relationship was quadratic, where, in low‐income areas, there was a positive relationship between street‐tree density and feeding migratory birds (Table 2, Fig. 4). However, as street‐tree density increased, the relationship changed to a negative slope (Fig. 4).

**Table 2:** Model‐selection results of three model sets relating migratory, year‐round, or total‐feeding bird density (dependent variables) to eight street‐tree diversity, density, or size attribute variables (independent variables), standardized per 1 km of survey route, within 36 residential communities throughout Los Angeles.

Parameter	Migratory			Year‐round			Total		
	∆AIC	β	β*^2^*	∆AIC	β	β*^2^*	∆AIC	β	β*^2^*
Intercept	27.79	6.49		7.79	3.82		19.81	10.31	
Street‐tree diversity									
Street‐tree species richness	29.03	1.09	1.01	4.80	1.40^†^	0.98	20.72	1.04	0.99
Street‐tree Shannon diversity‡	20.88	3.94^†^	0.61	9.79	1		18.88	0.72^†^	
Street‐tree density and size									
Total street‐tree *n*	0	1.07^†^	0.99^†^	4.63	1.03^†^	0.99	0	1.04^†^	0.99^†^
Native street‐tree *n*	22.84	1.09^†^	1	7.18	1.06^†^	0.99	15.02	1.08	0.99
Nonnative street‐tree *n*	2.66	1.08^†^	1^†^	5.69	1.03^†^	1	2.68	1.05^†^	0.99^†^
Total street‐tree basal area (m^2^)	14.12	1.02^†^	1	0	1.02^†^	0.99^†^	5.46	1.02^†^	0.99
Native street‐tree basal area (m^2^)	22.14	1.04	0.99	6.91	1.06	0.99	14.74	1.05^†^	0.99
Nonnative street‐tree basal area (m^2^)	16.45	1.02^†^	0.99	1.82	1.02^†^	0.99^†^	7.92	1.02^†^	0.99^†^

Notes

In addition to modeling all street trees combined within survey locations (total), we grouped tree density and size variables depending on whether street trees were native or nonnative to explore whether tree origin was an important predictor of feeding bird density. We fitted all models using a generalized linear modeling framework with a negative‐binomial error distribution, and we ranked models using Akaike’s Information Criterion (AIC). A ∆AIC of zero indicated the best‐supported model within a set, whereas values >2 suggested less support. We fitted all models, except for the intercept‐only model and the Shannon diversity for year‐round and total birds, using a quadratic term to account for hump‐shaped relationships prevalent in our data. We display the coefficient estimate (β) for both the fitted variable and its quadratic term and indicate the significance of a coefficient estimate with the dagger symbol (†). Further, as the negative‐binomial error distribution requires a log‐link transformation to estimate parameters, we display the β estimates on the original scale (i.e., exponentiated) for better interpretability. β estimates < 1 indicate negative relationships. The β estimate for the intercept represents the mean of the response variable, whereas the other coefficient estimates can be interpreted as follows; an increase in the independent variable by one unit would result in an increase (or decrease, note the quadratic formula required) of the response variable by a factor of the coefficient value.

‡ Not standardized to 1 km of walking route.

The top‐fitting model describing year‐round feeding bird density was the total‐tree basal area (Table 2, Fig. 4). This model was competitive with the nonnative tree basal area (∆AIC = 1.82, Table 2), but was superior to the intercept‐only model (∆AIC = 7.79, Table 2). Similar to the relationship with street‐tree density, the relationship was quadratic (Fig. 4). In low‐income areas, there was a positive relationship between the street‐tree basal area and year‐round feeding birds. Conversely, in affluent communities, the relationship shifted to negative as street trees covered more area (Fig. 4).

When relating all feeding birds (i.e., migratory and year‐round species combined) to street‐tree attributes, street‐tree density was again the top predictor variable (Table 2, Fig. 4). The change in the AIC value from the best‐fitting model to the second‐best model was 2.68, and the ∆AIC to the intercept‐only model was 19.81 (Table 2, Fig. 4). Further, the relationship was quadratic and nearly identical to the relationship between migratory birds and street‐tree density (Fig. 4). We did not find support that native street‐tree density or size were related to feeding‐bird density at the extent of our walking routes within LA neighborhoods (Table 2).

### Objective #3: foraging preferences of birds among both native and nonnative street trees

Both migratory and year‐round birds foraged on particular street trees in unequal proportions than they were available throughout the cityscape (χ^2^ = 34.44, *P* = 0.05 and χ^2^ = 46.59, *P* = 0.01, respectively). The most selective foraging migratory bird species were the Townsend’s Warbler (χ^2^ = 67.23, *P* < 0.01) and the Ruby‐crowned Kinglet (χ^2^ = 61.06, *P* < 0.01), whereas the most selective foraging year‐round bird species were the Lesser Goldfinch (χ^2^ = 94.58, *P* < 0.01), the Anna’s Hummingbird (χ^2^ = 82.64, *P* < 0.01), the House Finch (χ^2^ = 72.59, *P* < 0.01), and the Bushtit (χ^2^ = 70.04, *P* < 0.01). Of the seven species in which we had enough data for analysis, only the Yellow‐rumped Warbler foraged on street‐tree species in similar proportions to their availability, suggesting this species displays a wide breadth of foraging plasticity throughout the LA urban ecosystem during the winter months (χ^2^ = 25.79, *P* = 0.21).

**Fig. 5:**
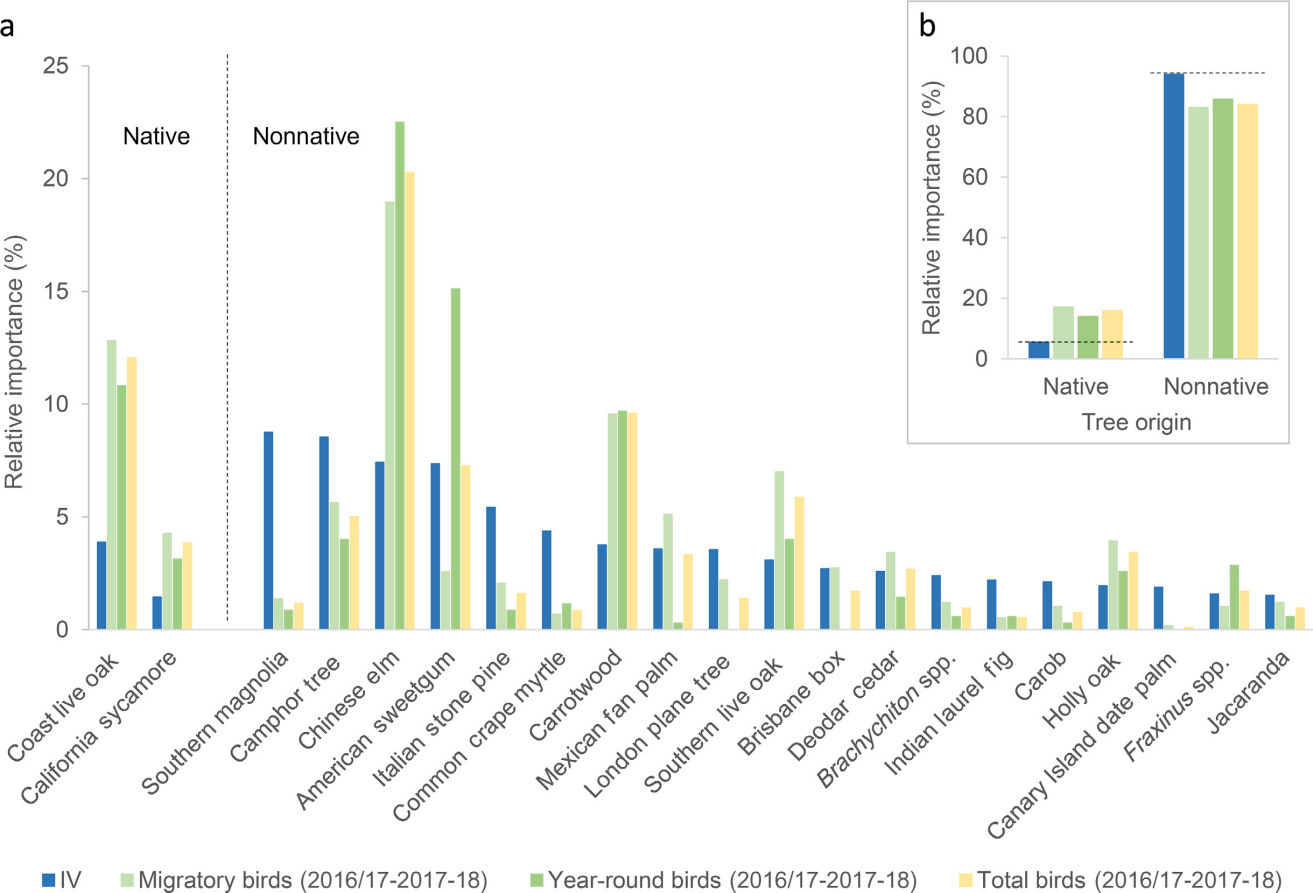
(a) Relative importance values of common street‐tree species (IV), grouped by whether they were native or nonnative in geographic origin, and the proportional use of native and nonnative trees by migratory, year‐round, and total birds (five migratory and five year‐round species combined) during the 2016–2017 and 2017–2018 winter field seasons throughout Los Angeles. (b) Inset figure depicts the relative importance values of grouped native and nonnative street‐tree species, and the proportional use of native and nonnative trees species by migratory, year‐round, and total birds. The street‐tree importance values represent a tree species’ or tree group’s availability as a foraging substrate to birds. Bars depicting bird‐foraging proportion that are greater than street‐tree importance values (horizontal dashed lines provided for reference in inset) suggest bird‐feeding preference, whereas bars below street‐tree importance values suggest bird‐feeding avoidance.

In general, we observed differences in foraging preference and aversion when comparing feeding patterns by birds on native and nonnative street trees (Table 3, Fig. 5a). Migratory and year‐round birds preferred foraging on native trees (preference index [PI] = 11.60 and 8.51, respectively) while avoiding nonnative trees (PI = −11.03 and −8.22, respectively, Table 3, Fig. 5b). The observed patterns of feeding preference equated to migratory and year‐round birds using native street trees, represented by the coast live oak and the California sycamore, 312% and 255% more than their availability throughout the cityscape (Table 3, Fig. 5b). Building on this finding, the coast live oak had one of the highest preference values by migratory and year‐round birds (PI = 8.92 and 6.94, respectively), whereas the California sycamore was lower (PI = 2.83 and 1.70, respectively, Table 3). When comparing patterns of use vs. availability of the two native tree species, individually, migratory and year‐round birds used both the coast live oak and the California sycamore in higher proportions (>200%) than their availability (Appendix S1: Table S4, Fig. 5). Migratory or year‐round birds did not use the three other native street‐tree species that we encountered (Appendix S1: Table S4).

**Table 3:** Street‐tree species preference (positive) and aversion (negative) values for year‐round, migratory, total (year‐round and migratory combined), and seven bird species throughout the Los Angeles urban forest.

Tree species	Year‐round	Migratory	Total	RCKI	TOWA	YRWA	ANHU	BUSH	HOFI	LEGO
Native										
Coast live oak	6.94	8.92	8.18	15.52	9.16	6.67	16.12	15.27	−1.80	−3.88
California sycamore	1.70	2.83	2.40	2.29	5.81	2.67	−1.44	2.82	0.65	3.48
Nonnative										
Southern magnolia	−7.90	‐7.39	−7.58	−8.75	−8.75	−6.41	−8.75	−8.75	−5.63	−8.75
Camphor tree	−4.56	−2.92	−3.53	−4.82	−1.30	−2.10	−5.22	−4.29	−5.42	−3.63
Chinese elm	15.09	11.52	12.86	13.47	23.01	6.95	−7.42	12.44	22.79	25.36
American sweetgum	7.75	−4.79	−0.10	−7.35	−7.35	−2.95	−4.02	−5.94	18.69	33.63
Italian stone pine	−4.58	−3.38	−3.83	−1.70	−1.08	−4.26	−2.10	−5.43	−3.35	−5.43
Common crape myrtle	−3.23	−3.69	−3.52	−4.37	−4.37	−3.20	−4.37	−2.24	−4.37	−4.37
Carrotwood	5.93	5.80	5.85	4.45	3.49	6.80	22.91	11.85	−3.76	−3.76
Mexican fan palm	−3.30	1.54	−0.28	−3.58	−3.58	5.21	−3.58	−3.58	−2.54	−3.58
London plane tree	−3.54	−1.32	−2.15	−2.05	−2.09	−0.61	−3.54	−3.54	−3.54	−3.54
Southern live oak	0.90	3.91	2.79	9.60	4.16	1.31	−3.08	4.72	−3.08	0.19
Brisbane box	−2.70	0.03	−1.00	−1.21	−2.70	0.52	−2.70	−2.70	−2.70	−2.70
Deodar cedar	−1.15	0.83	0.09	2.65	7.57	−0.82	−2.58	0.97	−2.58	−2.58
*Brachychiton* spp.	−1.82	−1.19	−1.43	−1.64	−2.39	−0.63	0.95	−2.39	−1.35	−2.39
Indian laurel fig	−1.62	−1.68	−1.66	−1.45	−2.19	−1.90	−2.19	−1.48	−2.19	−2.19
Carob	−1.83	−1.09	−1.36	−2.11	−2.11	−0.94	−2.11	−2.11	−2.11	−2.11
Holly oak	0.62	1.98	1.48	3.28	2.41	1.29	−1.94	4.44	−1.94	−1.94
Canary Island date palm	−1.87	−1.70	−1.76	−1.87	−1.87	−1.58	−1.87	−1.87	−1.87	−1.87
*Fraxinus* spp.	1.26	−0.56	0.12	−0.84	1.31	−0.71	−1.59	−1.59	8.83	−1.59
Jacaranda	−0.95	−0.32	−0.55	0.72	−0.07	−0.64	5.15	−1.51	−1.51	−1.51

Note

RCKI, Ruby‐crowned Kinglet; TOWA, Townsend’s Warbler; YRWA, Yellow‐rumped Warbler; ANHU, Anna’s Hummingbird; BUSH, Bushtit; HOFI, House Finch; and LEGO, Lesser Goldfinch.

In contrast, migratory and year‐round birds used the most common 19 nonnative street trees as foraging substrates 12% and 9% less than their availability, respectively (Fig. 5b). Nevertheless, our analysis did indicate a preference of birds to select nonnative street trees (Table 3, Fig. 5, Appendix S1: Table S4). The Chinese elm had the highest PI of all street trees by both migratory and year‐round birds (PI = 11.52 and 15.09), followed by the carrotwood (*Cupaniopsis anacardioides*, PI = 5.80 and 5.93), southern live oak (*Quercus virginiana*, PI = 3.91 and 0.90), and holly oak (*Quercus ilex*, PI = 1.98 and 0.62, Table 3).

Overall, migratory and year‐round birds used seven and six nonnative street‐tree species, respectively, in higher proportion than their availability (Appendix S1: Table S4, Fig. 5). All other nonnative street trees, which included approximately 90 species, family groups, or unknown individuals, were generally avoided by feeding birds (Appendix S1: Table S4). The highest proportional use of nonnative street trees by migratory birds was the Chinese elm (255% more than it was available), followed by the carrotwood (254%), southern live oak (227%), and holly oak (202%; Fig. 5, Appendix S1: Table S4). Year‐round birds used the Chinese elm and carrotwood in higher proportion than they were available throughout the cityscape (303% and 258%, respectively), followed by American sweetgum (205%), and *Fraxinus* spp. (180%; Fig. 5, Appendix S1: Table S4).

## Discussion

Given the pace and dominance of urbanization throughout the globe, understanding how to best manage and conserve biodiversity within city limits is a paramount challenge (Aronson et al. 2017, Lepczyk et al. 2017a). While there are initiatives in metropolises throughout the world to improve environmental quality within cities, understanding the ecology of street trees and birds has mostly been overlooked (with exceptions, see Tzilkowski et al. 1986, Young et al. 2007, and Shackleton 2016). Our results provide strong support that street trees have clear and positive value as foraging habitat to birds and thus are a critical resource for promoting urban avifauna. We found that across a socioeconomic gradient throughout LA, feeding bird density was positively associated with increases in density and size of street trees, especially in low‐ and medium‐income communities. Further, our study provided clear evidence for the positive benefit of two commonly planted native street‐tree species and a few nonnative tree species as foraging substrates for feeding birds. LA is located within a biodiverse region with avifauna abundant at the edges of the metropolis (Higgins et al. 2019). However, it is likely far more difficult for birds to persist in the most urbanized portions of the city (Blair 1996, McKinney 2006). Our findings indicate that planting and maintaining street trees within the boundaries of the metropolis will likely provide a substantial benefit to feeding birds.

Studies in other areas of the world have also indicated the importance of street trees to avian communities in urban ecosystems (Tzilkowski et al. 1986, Fernandez‐Juricic 2009, Shackleton 2016, De Castro Pena et al. 2017). For example, in the cities of Belo Horizonte, Brazil, and Madrid, Spain, bird species diversity was positively related to a diverse and dense street‐tree population (Fernandez‐Juricic 2009, De Castro Pena et al. 2017). In the towns of Amherst, Massachusetts, and Grahamstown, South Africa, the diversity of bird species occurring on streets increased with both the size (DeGraaf and Wentworth 1986) and the number of street‐tree species (Shackleton 2016). In contrast to the studies in Brazil and South Africa, we did not find associations between street‐tree richness and diversity and the bird response variables of our study. However, our research uncovered clear relationships with street‐tree density and size and feeding‐bird density, which supports findings from Spain (Fernandez‐Juricic 2009) and New England (DeGraaf and Wentworth 1986). A notable pattern of our results was the consistent humped‐shaped relationship between feeding‐bird density and street‐tree density and size. We found support that increases in street‐tree density and size in low‐income communities positively benefits feeding birds. However, the relationship shifted to negative in affluent areas. Affluent zones of our study system had far more vegetation in private yards than low‐income areas, which is a similar pattern to other studies in LA (Clarke et al. 2013). The abundance of vegetation in private yards may have provided additional habitat that attracted feeding birds from street trees (Lerman and Warren 2011, Belaire et al. 2014). Nevertheless, our findings underscore the critical importance to birds of planting and maintaining street trees in sections of the city that are lacking.

Throughout LA, we found that street trees and feeding‐bird density were far less in lower‐income than affluent communities. Our finding reaffirms support for the luxury‐effect hypotheses, which was apparent in our system in low‐ and medium‐income communities (Landry and Chakraborty 2009, Clarke et al. 2013). In LA, Clarke et al. (2013) studied vegetation cover and diversity in a variety of land‐use types throughout the residential areas of the city. Their study found that herbaceous and perennial vegetation was positively associated with income. However, they did not find support that tree diversity followed a similar pattern. Instead, the age of building development was the strongest predictor, with older developments having higher tree diversity (Clarke et al. 2013). Similar to Clarke et al. (2013), we did not find differences in the richness of street trees planted in low‐ and high‐income communities. However, our study revealed apparent differences in the density and size of street trees, which is similar to patterns seen in other cities (e.g., Tampa Bay [Landry and Chakraborty 2009], the Eastern Cape of South Africa [Kuruneri‐Chitepo and Shackleton 2011], and New York City [Schroeter 2017]). Further, we found that the differences in street‐tree density and basal area throughout LA also influenced the density of feeding birds. In addition to fewer and smaller street trees, our study indicated that low‐income residential communities of LA harbor a depauperate bird community, which is similar to patterns from other large cities (e.g., Phoenix, Arizona; Lerman and Warren 2011).

While our findings point out deficiencies in urban conservation throughout LA, our results also provide clear evidence for potential improvement. In lower‐income communities, we found that even small increases in the density and size of street trees is positively associated with a higher density of feeding birds. These results also hold for locations in LA far from protected areas, suggesting that street trees and birds are a viable target for improving conservation within urban ecosystems. Thus, initiatives to continue promoting trees in areas of a city lacking in street‐tree cover will likely have the most significant benefit to urban biodiversity conservation. One such effort, the Million Trees Initiative, has worked to plant trees in locations of LA with low tree density (McPherson et al. 2011). While such initiatives are designed to continue planting and maintaining street trees, tracking the success and long‐term viability of planted trees remains a challenge (Dudek 2018). Nevertheless, our results add an extension to the importance of supporting work such as the Million Trees Initiative as well as municipal urban forest programs, including up‐to‐date inventory and detailed information on tree planting needs.

In addition to the importance of street‐tree density and size as predictors of feeding bird density, our study provided an assessment of the value of over 100 street‐tree species (or family groups) to feeding birds throughout LA. We infrequently encountered nearly 80% of tree species in surveys (<1.5% IV), and thus, we treat assessments of the value of the uncommon species with caution. Nevertheless, the most important trees for feeding birds in our study system were a mixture of native and nonnative trees. While other studies have documented the importance of native and nonnative vegetation in urban areas to birds (e.g., Shackleton 2016, Narango et al. 2017, 2018), there were a few notable patterns within our system, including the role of trees in the genus *Quercus*. Oak trees of our study, one native and two nonnatives,– were nearly unparalleled in their use by feeding birds. Throughout the world, trees in the genus *Quercus* are valuable in providing numerous resources for wildlife, including as feeding substrate (Graber and Graber 1983, Rodewald and Abrams 2002) and breeding habitat (Parmain and Bouget 2018). Further, in eastern North America, oaks have some of the highest diversity and abundance of insects when compared with other common trees (Tallamy and Shropshire 2009).

Indeed, the importance of insect prey to feeding birds is becoming apparent in urban ecosystems. In the suburbs of Washington, D.C, plants with high insect food abundance positively benefited foraging and nesting success for the Carolina Chickadee (*Poecile carolinensis* Narango et al. 2017, 2018), while in Dunedin, New Zealand, the native Silvereye (*Zosterops lateralis*) foraged on trees with higher arthropod prey availability (Waite et al. 2013). Local (or native) tree species to a region that are planted in a cityscape have been suggested to harbor higher levels of invertebrate prey available to birds than nonindigenous species (Bhullar and Majer 2000). We did not measure food availability of street trees in our system. Further, our foraging behavioral data indicated similar foraging success among tree species (Appendix S1: Table S5). Nevertheless, our findings of the exceptionally high use of oaks by feeding birds may be due to the important role of oaks in urban ecosystems in structuring a diverse food web. Further, our findings suggest potentially an important functional similarity between native and nonnative oaks to feeding birds in urban ecosystems.

Other important tree species of our study for feeding birds included a sycamore (genus: *Platanus*), an elm (genus: *Ulmus*), and ash (genus: *Fraxinus*). Elsewhere in the world, elm and ash trees are valuable resources to feeding migratory birds (Wood et al. 2012), while sycamore trees provide valuable habitat for birds and other animals (Gabbe et al. 2002, Cudworth and Koprowski 2011). Our initial predictions were that native trees would be superior to nonnatives, and we did find strong support for this for the two most common native tree species of our study. However, we were surprised to find birds preferred a handful of nonnative species, even though studies in other urban areas have documented similar patterns (Gray and van Heezik 2016, Shackleton 2016).

Throughout the world, there has been considerable interest and debate about whether to promote native or nonnative trees in urban forests (Kendle and Rose 2000). Some studies illustrate the clear positive benefit of native plants to wildlife (e.g., Ikin et al. 2013, Narango et al. 2017, 2018), while others highlight the value of nonnative vegetation to urban biodiversity (e.g., DeGraaf 2002, Gray and van Heezik 2016, Shackleton 2016). For example, in South Africa, Shackleton (2016) found that nesting birds were more common in native than nonnative street trees. However, the study also noted the importance of nonnative street trees to native mistletoe (Shackleton 2016). In Dunedin, New Zealand, native and exotic birds fed on both native and nonnative trees (Gray and van Heezik 2016). Further, Gray and van Heezik (2016) found that nonnative trees provide food resources outside of the typical timing of native tree phenological events (e.g., berry and seed production). This finding suggests urban areas with nonnative vegetation may provide food resources outside of the typical seasonal pattern of adjacent natural areas. We also found that birds fed on a variety of native and nonnative tree substrates, including leaf surfaces, flowers, and fruits (Appendix S1). Having a variety of food resources available to birds in urban ecosystems throughout the annual cycle may be necessary when considering the effects of climate change on plant and food resource phenology, which in turn may influence bird utilization of a habitat (Wood and Pidgeon 2015).

Our results suggest that if promoting street trees to attract birds is a goal, there are likely numerous factors, in addition to geographical origin, to consider when making decisions about which trees to plant and promote (Kendle and Rose 2000, Sjöman et al. 2016). For example, LA is situated in an arid biome, and few native trees naturally occur in the region that would be suitable for planting along a street. LA has two of the most common native species of our study, the coast live oak and the California sycamore, planted throughout a handful of sections of the metropolis. However, over‐planting each tree could lead to problems. For example, the fungal pathogen Dutch elm disease decimated mature elm trees in many cities throughout the United States (Schlarbaum et al. 1997). Currently, the emerald ash borer beetle (*Agrilus planipennis*) is devastating ash trees throughout the midwestern and eastern United States (Poland and McCullough 2006), and in southern California, the South American palm weevil (*Rhynchophorus palmarum*) is currently infesting palm trees (Arecaceae) throughout the region (Hoddle 2019). There are current and potential threats already in the LA area, such as the invasive polyphagous shot‐hole borer beetle (*Euwallacea* spp.) and the gold‐spotted oak borer beetle (*Agrilus auroguttatus*), which can infest and kill coast live oak and California sycamore trees (Coleman et al. 2011, Kallstrand 2016). Such threats are behind the justification for the 10‐20‐30 rule, which states that urban tree populations should be no more than 10% of a particular species, 20% of a particular genus, or 30% of a particular family (Santamour 1990). While the 10‐20‐30 rule has been critiqued (Richards 1993, Raupp et al. 2006), having a diverse street‐tree canopy has been the target of many urban areas for providing resilience in the face of potential threats (Kendal et al. 2014, McPherson et al. 2016). Thus, lining streets with the two common native species of the LA region in a homogenous fashion likely raises the risk of possible threats. While there were three other native tree species that we encountered in our study, we could not accurately ascertain their value to feeding birds because these trees were so uncommon.

In more mesic portions of the world, where native tree diversity is higher in locations adjacent to cities, relying more on native tree species that are suitable for urban environments (e.g., tolerance to air pollution; Grote et al. 2016) may be an appropriate strategy when considering planting street trees (Jenerette et al. 2016). However, this may not be optimal for a city such as LA, or other cities in arid regions of the world with relatively poor tree diversity in lowland areas outside the city boundaries (Avolio et al. 2019). Thus, for many municipalities, nonnative street‐tree species likely need to be considered when thinking about a resilient urban forest canopy, which is a similar conclusion for cities elsewhere in the world (Sjöman et al. 2016). Extending this, there are numerous obstacles urban planners must contend with when considering the longevity of urban forests (Pretzsch et al. 2017). For example, when focusing on climate change, climate‐adapted trees may be a suitable strategy when weighing the needs of urban residents and wildlife (Jenerette et al. 2016, Lanza and Stone 2016). Our findings suggest that while there are indeed select nonnative street‐tree species that provide apparent benefits to feeding birds, many appear to be poor habitat. Thus, careful study of the value of a street‐tree species to feeding birds, or other wildlife (e.g., Bhullar and Majer 2000), and considering the other benefits a tree species provides to a city, is necessary for choosing optimal species to promote, especially if conservation is a goal.

Considering our research, we offer the following suggestions for managing street trees to benefit urban avifauna:

1. *Plantings*: cities must identify critical zones that are lacking in street‐tree density. While numerous factors may contribute to a lack of street‐tree density, our results, and those of others, suggest this will likely occur in lower‐income communities (Landry and Chakraborty 2009, Schroeter 2017).
2. *Incentivize maintenance*: once cities identify zones that are lacking in street‐tree density, promoting, planting, and maintaining street trees should be a goal. Many municipalities are already well‐aware of #1 and working to address #2 (e.g., Pincetl 2010). However, this is a difficult task since many units in lower‐income communities are often not owner‐occupied. Thus, there may be less of an incentive to encourage the growth of a street tree in front of the property (Landry and Chakraborty 2009). In these cases, cities should work to incentivize street‐tree care to the property owners or renters or provide public resources to promote the longevity of planted street trees.
3. *Street‐tree density targets*: If cities plant and maintain trees, our results suggest a target of approximately 40–120 street trees/1 km of street will likely attract feeding birds. We note that, in our system, there were few residential study areas with <40 trees/1 km. Thus, our confidence in estimates at these ranges is low. The 40–120 numbers refer to trees on both sides of a street and can likely be halved if only considering one side of a street. Some municipalities may have zones where this is not feasible. If so, our study suggests that even modest increases in street‐tree density – coupled with careful consideration of tree species ‐ will likely provide valuable habitat to feeding birds.
4. *Long‐term maintenance*: long‐term maintenance of street trees and the encouragement of their growth is imperative to maximize the benefit to urban avifauna. Our results suggest that targeting up to approximately 125 m^2^ of the area covered by street trees per 1 km will likely attract feeding birds.
5. *Inventory*: many municipalities have inventories in place detailing information such as the location, size, date planted, health, and species of tree, for all street trees within city boundaries. Having a detailed street‐tree inventory is a critical step for municipalities to understand how to manage street trees based on a city’s needs, including providing assessments (the current study) and services to aid biodiversity (Dudek 2018). Further, detailed inventories allow for appropriate planning of diversity targets for street trees (Santamour 1990, McPherson et al. 2016).
6. *Native and nonnative trees*: our study indicates that the common native trees of our region, along with a handful of nonnative street trees can be beneficial to feeding birds. We do stress that the vast majority of nonnative trees in LA appear to provide little apparent benefit to the feeding birds of our study. Thus, our work suggests that careful consideration is required to determine the best street trees to plant and maintain if providing habitat for birds is a goal. If possible, municipalities should use available information (e.g., National Audubon Society 2019) coupled with careful study to identify which trees will provide essential services to both humans and birds.
7. *Value of studying feeding birds*: while there are numerous taxa of wildlife found in cities that likely utilize street trees (e.g., insects, birds, mammals), we suggest focusing attention on feeding birds. Birds are one of the most abundant and diverse wildlife taxa in most cities throughout the world (Lepczyk et al. 2017b). Further, they are relatively easy to study compared with other abundant taxa (e.g., insects; Bhullar and Majer 2000). A bird feeding on a tree substrate is an intricate and detailed ecological process that yields great information about which trees are beneficial to birds, and possibly other wildlife (Holmes and Robinson 1981, Gabbe et al. 2002, Wood et al. 2012). If municipalities already have tree inventories in place (see #5), a study needs to only focus on observing feeding birds on street trees in a given area over a given period, which can then be compared with the detailed street‐tree data similarly as this study. A unique component of LA’s avifauna are wintering migratory birds. In different urbanized locations of the world, a study such as ours could consider *en‐route* migratory birds (e.g., urban stop‐over locations, Amaya‐Espinel and Hostetler 2019) or breeding species (DeGraaf and Wentworth 1986). City personnel, arborists, students, volunteers, or citizen‐science initiatives can accomplish a study detailing the behavior of feeding birds on street trees.

## Supporting information

Appendix S1

## Data Availability

Data are available in the Dryad Digital Repository: https://doi.org/10.5061/dryad.qfttdz0d6
