# Supplementary material for: The importance of street trees to urban avifauna
Source: Ecol Appl. 2020 Jun 11;30(7):e02149. doi: 10.1002/eap.2149 (PMC7583466; doi:10.1002/eap.2149)
Supplement: Supplementary file 1 — Appendix S1 [file EAP-30-e02149-s001.pdf]

Supporting Information. Wood, E.M., and S. Esaian. 2020. The importance of street trees to urban avifauna. Ecological Applications.

Appendix S1. Extended methods, results, and discussion.

Table S1. Summaries of migratory study bird foraging observations.

Table S2. Summaries of year-round study bird foraging observations.

Table S3. Summaries of street tree density and size.

Table S4. Street-tree importance values and feeding bird observations for all street tree species.

Table S5. Summaries of migratory bird foraging behavior on nine common tree species.

Figure S1. Semivariograms of the residuals of response and independent variables.

Figure S2. Spearman's rho (ρ) correlation between 'reduced' and 'all' individual flock estimates.

Appendix S1

Extended Methods and Results

Spatial autocorrelation

A critical concern when designing a study across a spatially segregated gradient, such as the socioeconomic gradient in LA, are patterns of autocorrelation among survey locations (Legendre 1993). Issues with autocorrelation can violate the assumption of independence, which is a key assumption of many statistical analyses (Zuur et al. 2011). Before analysis, we tested for patterns of spatial dependence among our survey locations by computing a semivariogram analysis (Legendre and Fortin 1989) using the 'geoR' package in R (Ribeiro Jr and Diggle 2018). The semivariogram analysis calculated the degree of dissimilarity of the residuals of bird and tree variables (see statistical analysis for details on variables) between survey locations as a function of distance (Legendre and Fortin 1989). There were no issues with spatial dependence of our survey locations based on the semivariogram analysis, and therefore, we considered all survey locations as independent (Appendix S1: Fig. S1).

Flock-density estimation

Due to the flocking nature of a handful of year-round focal species (e.g., the Bushtit, *Psaltriparus minimus*, the Lesser Goldfinch, *Spinus psaltria*, and the House Finch, *Haemorhous mexicanus*), we opted to consider larger, homogenous flocks of these species as an $n = 1$. We employed the reduced method to avoid overinflating the ecological importance of a given tree on the movement and feeding patterns of a group of birds. To ascertain whether the 'reduced' method yielded similar results compared with including all individuals of the same species within a flock ('all'), we computed two analyses. First, we calculated a Spearman's rho (ρ)

correlation between the density of ‘reduced’ and ‘all’ year-round birds. The Spearman’s ρ value was 0.81, *p-value* < 0.01 (Appendix S1: Fig. S2). Further, we computed an ANOVA analysis of both ‘reduced’ and ‘all’ density measures among the three income groups of our study. Our intention with the ANOVA analysis was to compare similarities in effect sizes between response variables. We found that the ‘reduced’ measure yielded an *F*-value of 5.182, and indicated strong significant differences (*p-value* = 0.011) of year-round bird density among income groups (Table 1, in manuscript). We found nearly identical effect-sizes for the ‘all’ measure, *F*-value of 5.182, *p-value* = 0.011. Our results support that our ‘reduced’ treatment of the year-round flock density values yields similar effects to the ‘all’ measure.

Street-tree phenology and foraging-bird behavior

To understand patterns in bird feeding depending on the phenology of tree species throughout the winter months, we documented the dominant phenophase of a street tree in which we completed a foraging observation. We created the following categories to describe tree phenology: (a) ‘leafed out’, full leaf canopy with < 25% of visible flowers, seeds, fruits, or senescing leaves; (b) ‘seeding’, > 75% of a tree’s canopy containing visible seeds or nuts; (c) ‘flowering’, > 75% of a tree’s canopy containing visible flowers; (d) ‘fruiting’, > 75% of canopy containing visible fleshy fruit; (e) ‘leaf senescence’, > 75% of canopy with senescing leaves, or (f) ‘leafless’, a winter deciduous or dead tree, with no visible live leaves.

In general, birds exhibited variable feeding patterns based on differences in phenology of street trees. When examining the dominant phenological stage of both native and non-native trees in which we observed migratory bird foraging, trees were primarily leafing (*n* = 192, 35.75%), followed by seeding (*n* = 138, 25.70%), flowering (*n* = 80, 14.90%), fruiting (*n* = 51,

9.50%), leaf senescence (*n* = 72, 13.41%), or leafless (*n* = 4, 0.74%). Of the 587 observed foraging observations and 2246 attack maneuvers of migratory birds, 47.06% of attacks were on leaf surfaces (*n* = 1057), 20.30% were aerial attacks (*n* = 456), 14.95% were on woody surfaces (*n* = 336), 12.37% were on flowers (*n* = 278), and 5.29% were on fruit surfaces (*n* = 119). We recorded migratory bird feeding in 49 other individual trees where we did not record tree-phenology stage. For year-round birds, we did not record detailed attack maneuvers on a tree substrate in which a bird was feeding, but we did record the dominant phenology stage of each tree in which we made a bird observation. Of the 351 observations, year-round birds primarily fed in trees that were seeding (*n* = 175, 54.86%), leafing (*n* = 64, 20.06%), flowering (*n* = 53, 20.06%), fruiting (*n* = 14, 4.39%), or leafless (*n* = 14, 4.39%). We recorded resident bird feeding in 32 other tree species where we did not record dominant tree phenology.

The foraging behavior of birds can be used as an indirect proxy for the availability of invertebrate prey items on trees species (Lovette and Holmes 1995). We found that the attack index of birds, which is defined as a ratio of the number of attacks to search maneuvers, standardized to a similar time of observation (Lovette and Holmes 1995, Oyugi et al. 2012, Wood et al. 2012), was similar among nine common tree species in which we had sufficient foraging data for analysis (Appendix S1: Table S5). We performed an ANOVA analysis to test whether there were differences in the mean attack rate among tree species, which revealed possible differences ($F_{8, 267} = 1.96$, *p-value* = 0.05). However, a subsequent Tukey's HSD test yielded no significant differences in the attack index among tree species. Attacks, ignoring search efforts, were more variable among tree species ($F_{8, 267} = 3.31$, *p-value* < 0.01, Appendix S1: Table S5). However, after computing a Tukey's HSD test, the only difference, based on a Bonferroni adjusted alpha value of 0.05/35 comparisons = 0.0014, was between the Camphor

Tree and the Italian Stone Pine (*p-value* = 0.0003). There were no differences in total search efforts among the nine common tree species ($F_{8, 267}$ = 1.71, *p-value* = 0.08).

Literature Cited

Legendre, P. 1993. Spatial autocorrelation: trouble or new paradigm? Ecology 74:1659–1673.

Legendre, P., and M.-J. Fortin. 1989. Spatial pattern and ecological analysis. Vegetatio 80:107–138.

Lovette, I. J., and R. T. Holmes. 1995. Foraging behavior of American Redstarts in breeding and wintering habitats: implications for relative food availability. The Condor 97:782–791.

Oyugi, J. O., J. S. Brown, and C. J. Whelan. 2012. Foraging behavior and coexistence of two sunbird species in a Kenyan woodland. Biotropica 44:262–269.

Ribeiro Jr, P. J., and P. J. Diggle. 2018. geoR: Analysis of geostatistical data.

Wood, E. M. M., A. M. M. Pidgeon, F. Liu, and D. J. J. Mladenoff. 2012. Birds see the trees inside the forest: the potential impacts of changes in forest composition on songbirds during spring migration. Forest Ecology and Management 280:176–186.

Zuur, A. F., E. N. Ieno, N. J. Walker, A. A. Saveliev, and G. M. Smith. 2011. Mixed-effects models and extensions in ecology with R. Springer US, New York, New York, USA.

Table S1. Foraging behavioral observations of five migratory bird species observed feeding on public street trees during two winter field seasons along a socioeconomic gradient of low (< $53,219 medium household income), medium ($53,220 - $70,719), and high-income residential communities (> $70,720) throughout Greater Los Angeles. Numbers indicate the total number of foraging observations, whereas values in superscript represent the cumulative number of seconds we observed a bird foraging. Numbers in superscript within parentheses indicate total attacks and total searches.

Common name	Scientific name	Socioeconomic group	2016 - 2017	2017 - 2018
Ruby-crowned Kinglet	*Regulus calendula*	Low	$8^{385\ (41,\ 163)}$	$7^{485\ (28,\ 203)}$
		Medium	$11^{575\ (85,\ 244)}$	$12^{1001\ (54,\ 309)}$
		High	$52^{4285\ (356,\ 1934)}$	$46^{2910\ (234,\ 1312)}$
Orange-crowned Warbler	*Vermivora celata*	Low	$5^{295\ (14,\ 46)}$	$2^{92\ (4,\ 21)}$
		Medium	$3^{566\ (39,\ 163)}$	0
		High	$4^{301\ (36,\ 110)}$	$9^{530\ (26,\ 161)}$
Yellow-rumped Warbler	*Setophaga coronata*	Low	$44^{2365\ (109,\ 681)}$	$22^{1349\ (56,\ 412)}$
		Medium	$44^{2248\ (115,\ 674)}$	$36^{1601\ (77,\ 488)}$
		High	$115^{7460\ (460,\ 1808)}$	$87^{5507\ (312,\ 1471)}$
Black-throated gray Warbler	*Setophaga nigrescens*	Low	$2^{168\ (18,\ 57)}$	0
		Medium	$1^{60\ (4,\ 17)}$	0
		High	$3^{191\ (19,\ 58)}$	$4^{517\ (27,\ 79)}$
Townsend's Warbler	*Setophaga townsendi*	Low	$3^{279\ (18,\ 95)}$	$6^{256\ (13,\ 70)}$
		Medium	0	$8^{750\ (41,\ 216)}$
		High	$20^{1455\ (92,\ 480)}$	$32^{2226\ (104,\ 602)}$

Table S2. Foraging behavioral observations of five year-round bird species observed feeding on public street trees during two winter field seasons along a socioeconomic gradient of low (< $53,219 medium household income), medium ($53,220 - $70,719), and high-income residential communities (> $70,720) throughout Greater Los Angeles. Numbers indicate the total number of foraging observations. We did not monitor individual foraging behavior of year-round species as observations were often of a stationary bird feeding.

Common name	Scientific name	Socioeconomic group	2016 - 2017	2017 - 2018
Allen's Hummingbird	*Selasphorus sasin*	Low	2	3
		Medium	6	2
		High	7	3
Anna's Hummingbird	*Calypte anna*	Low	2	0
		Medium	3	6
		High	10	9
Bushtit	*Psaltriparus minimus*	Low	20	20
		Medium	11	14
		High	30	46
Lesser Goldfinch	*Spinus psaltria*	Low	2	9
		Medium	7	4
		High	23	16
House Finch	*Haemorhous mexicanus*	Low	31	12
		Medium	10	8
		High	23	12

Table S3. Total observed street trees (tree *n*), average street-tree species diameter at breast height [tree DBH (cm)], total street-tree species basal area [tree basal area (m^2)], and relative values for tree *n*, tree basal area, and importance value for 108 street tree species, or families, throughout 36 Los Angeles study locations.

Tree species	*Scientific name*	Tree (*n*)	Tree DBH (cm)	Tree basal area (m^2)	Relative tree *n* (%)	Relative tree basal area (%)	Relative importance value (%)
Native							
Coast Live Oak	*Quercus agrifolia*	236	76.09	186	3.09	4.67	3.88
California Sycamore	*Platanus racemosa*	79	94.85	73.30	1.03	1.84	1.44
Engelmann Oak	*Quercus engelmannii*	8	47.63	1.77	0.10	0.04	0.07
California Bay Laurel	*Umbellularia californica*	5	38.10	0.75	0.07	0.02	0.04
California Black Walnut	*Juglans californica*	3	42.33	0.43	0.04	0.01	0.03
Non-native							
Southern Magnolia	*Magnolia grandiflora*	700	68.88	332	9.17	8.34	8.75
Common Crape Myrtle	*Lagerstroemia indica*	592	25.58	39.34	7.75	0.99	4.37
American Sweetgum	*Liquidambar styraciflua*	546	73.42	300.75	7.15	7.56	7.35
Camphor Tree	*Cinnamomum camphora*	530	86.95	404.18	6.94	10.16	8.55
Chinese Elm	*Ulmus parvifolia*	499	80.89	330.67	6.53	8.31	7.42
London Plane Tree	*Platanus × acerifolia*	368	54.02	90.14	4.82	2.27	3.54
Mexican Fan Palm	*Washingtonia robusta*	313	65.99	122.11	4.10	3.07	3.58

Carrotwood	*Cupaniopsis anacardioides*	311	66.99	136.83	4.07	3.44	3.76
Brisbane Box	*Lophostemon confertus*	274	52.26	72.42	3.59	1.82	2.70
Non-native, unknown		257	37.56	67.34	3.37	1.69	2.53
Brachychiton spp.	*Brachychiton* spp.	233	53.66	68.60	3.05	1.72	2.39
Holly Oak	*Quercus ilex*	211	47.09	44.46	2.76	1.12	1.94
Indian Laurel Fig	*Ficus microcarpa*	173	66.01	84.41	2.27	2.12	2.19
Pistacia spp.	*Pistacia* spp.	149	25.83	19.06	1.95	0.48	1.22
Jacaranda	*Jacaranda mimosifolia*	140	55.48	47.62	1.83	1.20	1.51
Carob	*Ceratonia siliqua*	136	84.38	97.14	1.78	2.44	2.11
Queen Palm	*Syagrus romanzoffiana*	129	38.79	19.15	1.69	0.48	1.09
Southern Live Oak	*Quercus virginiana*	110	117.69	188.16	1.44	4.73	3.08
Chinese Flame Tree	*Koelreuteria bipinnata*	95	52.24	35.85	1.24	0.90	1.07
Callery Pear	*Pyrus calleryana*	91	70.12	45.59	1.19	1.15	1.17
Canary Island Date Palm	*Phoenix canariensis*	91	112.82	101.36	1.19	2.55	1.87
Italian Stone Pine	*Pinus pinea*	91	221.62	384.74	1.19	9.67	5.43
Fraxinus spp.	*Fraxinus* spp.	82	90.42	83.45	1.07	2.10	1.59
Ulmus spp.	*Ulmus* spp.	81	72.37	48.15	1.06	1.21	1.14
Myrtaceae spp.	*Myrtaceae* spp.	67	49.17	14.32	0.88	0.36	0.62
Deodar Cedar	*Cedrus deodara*	64	167.48	171.88	0.84	4.32	2.58

Phanera spp.	*Phanera* spp.	64	32.94	8.64	0.84	0.22	0.53
Gold Medallion	*Cassia leptophylla*	59	66.73	26.55	0.77	0.67	0.72
Melaleuca spp.	*Melaleuca* spp.	52	122.21	83.16	0.68	2.09	1.39
Eucalyptus spp.	*Eucalyptus* spp.	51	88.70	39.25	0.67	0.99	0.83
Australian Willow	*Geijera parviflora*	46	32.41	5.18	0.60	0.13	0.37
Pink Trumpet Tree	*Handroanthus impetiginosus*	46	20.98	2.33	0.60	0.06	0.33
Canary Island Pine	*Pinus canariensis*	44	82.55	31.69	0.58	0.80	0.69
Prunus spp.	*Prunus* spp.	44	21.71	1.95	0.58	0.05	0.31
Japanese Zelkova	*Zelkova serrata*	32	19.05	1.04	0.42	0.03	0.22
California Fan Palm	*Washingtonia filifera*	31	90.13	20.80	0.41	0.52	0.46
Australian Pine Tree	*Casuarina equisetifolia*	30	103.29	32.30	0.39	0.81	0.60
California Peppertree	*Schinus molle*	27	59.74	11.11	0.35	0.28	0.32
Ficus spp.	*Ficus* spp.	26	36.15	3.14	0.34	0.08	0.21
Loquat	*Eriobotrya japonica*	26	36.63	3.56	0.34	0.09	0.21
Pittosporum spp.	*Pittosporum* spp.	20	18.42	0.62	0.26	0.02	0.14
Cercis spp.	*Cercis* spp.	19	84.89	15.26	0.25	0.38	0.32
Evergreen Pear	*Pyrus kawakamii*	17	23.91	1.01	0.22	0.03	0.12
Acacia spp.	*Acacia* spp.	16	46.04	3.14	0.21	0.08	0.14
Windmill Palm	*Trachycarpus fortunei*	15	58.42	4.50	0.20	0.11	0.15

Goldenrain Tree	*Koelreuteria paniculata*	13	15.63	0.35	0.17	0.01	0.09
Italian Cypress	*Cupressus sempervirens*	13	50.02	3.72	0.17	0.09	0.13
Podocarpus spp.	*Podocarpus* spp.	13	50.80	3.85	0.17	0.10	0.13
Chinese Tallow Tree	*Triadica sebifera*	12	16.93	0.46	0.16	0.01	0.08
Fern Pine	*Afrocarpus gracilior*	12	31.75	1.06	0.16	0.03	0.09
Olive	*Olea europaea*	12	64.56	4.42	0.16	0.11	0.13
Tulip Tree	*Liriodendron tulipifera*	12	25.40	0.61	0.16	0.02	0.09
Norfolk Island Pine	*Araucaria heterophylla*	11	63.5	4.24	0.14	0.11	0.13
Southern Silky Oak	*Grevillea robusta*	11	45.03	2.26	0.14	0.06	0.10
Aleppo Pine	*Pinus halepensis*	10	95.25	9.84	0.13	0.25	0.19
Catalpa spp.	*Catalpa* spp.	9	26.81	0.72	0.12	0.02	0.07
Cupressaceae spp.	*Cupressaceae* spp.	9	59.27	2.81	0.12	0.07	0.09
Ligustrum spp.	*Ligustrum* spp.	9	35.28	1.03	0.12	0.03	0.07
Silk Floss Tree	*Ceiba speciosa*	9	52.21	2.12	0.12	0.05	0.09
Brazilian Peppertree	*Schinus terebinthifolius*	8	47.63	1.57	0.10	0.04	0.07
Cajeput	*Melaleuca quinquenervia*	8	57.15	2.56	0.10	0.06	0.08
Chinaberry Tree	*Melia azedarach*	8	44.45	1.39	0.10	0.04	0.07
Cork Oak	*Quercus suber*	8	31.75	0.81	0.10	0.02	0.06
King Palm Tree	*Archontophoenix alexandrae*	8	26.99	0.52	0.10	0.01	0.06

Orange Tree	*Citrus X sinensis*	8	42.86	1.51	0.10	0.04	0.07
Desert Willow	*Chilopsis linearis*	7	34.47	0.95	0.09	0.02	0.06
Afghan Pine	*Pinus eldarica*	7	59.87	2.06	0.09	0.05	0.07
Betula spp.	*Betula* spp.	7	27.21	0.47	0.09	0.01	0.05
Chinese Fringe Tree	*Chionanthus retusus*	7	12.70	0.09	0.09	0	0.05
Persian Silk Tree	*Albizia julibrissin*	7	52.61	2.34	0.09	0.06	0.08
Gleditsia spp.	*Gleditsia* spp.	6	33.87	0.68	0.08	0.02	0.05
Lemon Tree	*Citrus limon*	6	27.52	0.42	0.08	0.01	0.04
Morus spp.	*Morus* spp.	4	34.93	0.47	0.05	0.01	0.03
Pygmy Date Palm	*Phoenix roebelenii*	4	25.40	0.20	0.05	0.01	0.03
Coast Redwood	*Sequoia sempervirens*	3	71.97	1.28	0.04	0.03	0.04
Avocado Tree	*Persea americana*	3	12.70	0.04	0.04	0	0.02
Ginkgo	*Ginkgo biloba*	3	29.63	0.27	0.04	0.01	0.02
Japanese Maple	*Acer palmatum*	3	12.70	0.04	0.04	0	0.02
Majesty Palm	*Ravenea rivularis*	3	25.40	0.15	0.04	0	0.02
Acer spp.	*Acer* spp.	2	19.05	0.06	0.03	0	0.01
Alnus spp.	*Alnus* spp.	2	38.10	0.23	0.03	0.01	0.02
Apricot Tree	*Prunus* spp.	2	19.05	0.06	0.03	0	0.01
Cook Pine	*Araucaria columnaris*	2	31.75	0.22	0.03	0.01	0.02

Erythrina spp.	*Erythrina* spp.	2	292.10	14.04	0.03	0.35	0.19
Guadalupe Palm	*Brahea edulis*	2	38.10	0.25	0.03	0.01	0.02
Sago Palm	*Cycas revoluta*	2	25.40	0.10	0.03	0	0.01
Pencil Cactus	*Euphorbia tirucalli*	2	25.40	0.10	0.03	0	0.01
Peppermint Tree	*Agonis flexuosa*	2	25.40	0.10	0.03	0	0.01
Black Locust	*Robinia pseudoacacia*	2	38.10	0.25	0.03	0.01	0.02
Spanish Dagger Yucca	*Yucca gloriosa*	2	25.40	0.10	0.03	0	0.01
Monterey Pine	*Pinus radiata*	1	12.70	0.01	0.01	0	0.01
American Sycamore	*Prunus armeniaca*	1	50.80	0.20	0.01	0.01	0.01
Chinese Hibiscus	*Hibiscus rosa-sinensis*	1	25.40	0.05	0.01	0	0.01
Fountain Palm	*Livistona chinensis*	1	38.10	0.11	0.01	0	0.01
Red Tip Photinia	*Photinia × fraseri*	1	12.70	0.01	0.01	0	0.01
Golden Shower Tree	*Cassia fistula*	1	38.10	0.11	0.01	0	0.01
Guava Tree	*Psidium guajava*	1	12.70	0.01	0.01	0	0.01
Oleander	*Nerium oleander*	1	12.70	0.01	0.01	0	0.01
Peach Tree	*Prunus persica*	1	25.40	0.05	0.01	0	0.01
Pecan Tree	*Carya illinoinensis*	1	76.20	0.46	0.01	0.01	0.01
Star Magnolia	*Magnolia stellata*	1	12.70	0.01	0.01	0	0.01
Tilia spp.	*Tilia* spp.	1	25.40	0.05	0.01	0	0.01

Tree of Heaven	*Ailanthus altissima*	1	25.40	0.05	0.01	0	0.01

Table S4. Relative street tree importance values (IV), and proportion of feeding observations of migratory, year-round, and total birds (migratory and year-round observations combined) during the 2016/2017 and 2017/2018 winter field seasons on 108 street tree species throughout 36 Los Angeles study locations. We define feeding as an observed feeding maneuver on a tree substrate (i.e., an attack directed at the bark, leaf, bud, flower, fruit, seed, or aerial maneuver within the limits of the tree canopy). Feeding proportion values higher than street tree IVs suggest foraging preference, whereas feeding proportion values lower than street tree IVs suggest foraging avoidance by migratory or year-round birds.

Tree species	*Scientific name*	IV	Migratory bird feeding (2016)	Migratory bird feeding (2017)	Year-round bird feeding (2016)	Year-round bird feeding (2017)	Total bird feeding (2016)	Total bird feeding (2017)
Native								
Coast Live Oak	*Quercus agrifolia*	3.88	10.79	15.13	10.16	11.59	10.56	13.79
California Sycamore	*Platanus racemosa*	1.44	4.13	4.43	2.67	3.66	3.59	4.14
Engelmann Oak	*Quercus engelmannii*	0.07	0	0	0	0	0	0
California Bay Laurel	*Umbellularia californica*	0.04	0	0	0	0	0	0
California Black Walnut	*Juglans californica*	0.03	0	0	0	0	0	0
Non-native								
Southern Magnolia	*Magnolia grandiflora*	8.75	2.22	0.37	1.60	0	1.99	0.23
Common Crape Myrtle	*Lagerstroemia* spp.	4.37	0.95	0.37	0	2.44	0.60	1.15
American Sweetgum	*Liquidambar styraciflua*	7.35	2.22	2.95	15.51	14.63	7.17	7.36
Camphor Tree	*Cinnamomum camphora*	8.55	4.44	7.01	2.67	5.49	3.78	6.44

Chinese Elm	*Ulmus parvifolia*	7.42	17.14	21.03	20.32	25	18.33	22.53
London Plane Tree	*Platanus × acerifolia*	3.54	1.90	2.58	0	0	1.20	1.61
Mexican Fan Palm	*Washingtonia robusta*	3.58	6.03	4.06	0.53	0	3.98	2.53
Carrotwood	*Cupaniopsis anacardioides*	3.76	9.52	9.59	6.42	13.41	8.37	11.03
Brisbane Box	*Lophostemon confertus*	2.70	2.86	2.58	0	0	1.79	1.61
Non-native, Unknown		2.53	5.71	1.48	9.09	0	6.97	0.92
Brachychiton spp.	*Brachychiton* spp.	2.39	0.95	1.48	1.07	0	1	0.92
Holly Oak	*Quercus ilex*	1.94	4.13	3.69	2.67	2.44	3.59	3.22
Indian Laurel Fig	*Ficus microcarpa*	2.19	0.95	0	1.07	0	1	0
Pistacia spp.	*Pistacia* spp.	1.22	0	0	0	0	0	0
Jacaranda	*Jacaranda mimosifolia*	1.51	0.95	1.48	1.07	0	1	0.92
Carob	*Ceratonia siliqua*	2.11	0.63	1.48	0.53	0	0.60	0.92
Queen Palm	*Syagrus romanzoffiana*	1.09	0.32	0	0	0	0.20	0
Southern Live Oak	*Quercus virginiana*	3.08	9.21	4.43	4.28	3.66	7.37	4.14
Chinese Flame Tree	*Koelreuteria bipinnata*	1.07	1.27	0.37	0	1.83	0.80	0.92
Callery Pear	*Pyrus calleryana*	1.17	0.95	0	0.53	0	0.80	0
Canary Island Date Palm	*Phoenix canariensis*	1.87	0.32	0	0	0	0.20	0
Italian Stone Pine	*Pinus pinea*	5.43	2.22	1.85	1.60	0	1.99	1.15
Fraxinus spp.	*Fraxinus* spp.	1.59	0.63	1.48	3.21	2.44	1.59	1.84

Ulmus spp.	*Ulmus* spp.	1.14	0.63	0	1.60	0	1	0
Myrtaceae spp.	*Myrtaceae* spp.	0.62	0	0	0	0	0	0
Deodar Cedar	*Cedrus deodara*	2.58	2.22	4.80	1.60	1.22	1.99	3.45
Phanera spp.	*Phanera* spp.	0.53	0	0	1.07	0	0.40	0
Gold Medallion	*Cassia leptophylla*	0.72	0	0	0	0	0	0
Melaleuca spp.	*Melaleuca* spp.	1.39	3.49	4.06	2.14	8.54	2.99	5.75
Eucalyptus spp.	*Eucalyptus* spp.	0.83	1.27	1.48	2.67	0.61	1.79	1.15
Australian Willow	*Geijera parviflora*	0.37	0.32	0	0	0	0.20	0
Pink Trumpet Tree	*Handroanthus impetiginosus*	0.33	0	0	0	0	0	0
Canary Island Pine	*Pinus canariensis*	0.69	0	0.37	0	0	0	0.23
Prunus spp.	*Prunus* spp.	0.31	0	0	0	0	0	0
Japanese Zelkova	*Zelkova serrata*	0.22	0	0	0	0	0	0
California Fan Palm	*Washingtonia filifera*	0.46	0	0	0	0	0	0
Australian Pine Tree	*Casuarina equisetifolia*	0.60	0	0	1.60	1.22	0.60	0.46
California Peppertree	*Schinus molle*	0.32	0	0	0	0	0	0
Ficus spp.	*Ficus* spp.	0.21	0	0	0	0	0	0
Loquat	*Eriobotrya japonica*	0.21	0	0	0	0	0	0
Pittosporum spp.	*Pittosporum* spp.	0.14	0	0	0	0	0	0

Cercis spp.	*Cercis* spp.	0.32	0	0	0	0	0	0
Evergreen Pear	*Pyrus kawakamii*	0.12	0	0	0	0	0	0
Acacia spp.	*Acacia* spp.	0.14	0	0	0	0	0	0
Windmill Palm	*Trachycarpus fortunei*	0.15	0	0	0	0	0	0
Goldenrain Tree	*Koelreuteria paniculata*	0.09	0	0	0	0	0	0
Italian Cypress	*Cupressus sempervirens*	0.13	0	0	0	0	0	0
Podocarpus spp.	*Podocarpus* spp.	0.13	0	0	0	0	0	0
Chinese Tallow Tree	*Triadica sebifera*	0.08	0	0	0	0	0	0
Fern Pine	*Afrocarpus gracilior*	0.09	0	0.74	0	0	0	0.46
Olive	*Olea europaea*	0.13	0.63	0.37	1.60	1.83	1	0.92
Tulip Tree	*Liriodendron tulipifera*	0.09	0	0	0	0	0	0
Norfolk Island Pine	*Araucaria heterophylla*	0.13	0	0	0	0	0	0
Southern Silky Oak	*Grevillea robusta*	0.10	0	0	0	0	0	0
Aleppo Pine	*Pinus halepensis*	0.19	0	0	0	0	0	0
Catalpa spp.	*Catalpa* spp.	0.07	0	0	0	0	0	0
Cupressaceae spp.	*Cupressaceae* spp.	0.09	0	0	0	0	0	0
Ligustrum spp.	*Ligustrum* spp.	0.07	0.32	0	0	0	0.20	0
Silk Floss Tree	*Ceiba speciosa*	0.09	0.32	0	0	0	0.20	0
Brazilian Peppertree	*Schinus terebinthifolius*	0.07	0	0	0	0	0	0

Cajeput	*Melaleuca quinquenervia*	0.08	0	0	0	0	0	0
Chinaberry Tree	*Melia azedarach*	0.07	0	0	0	0	0	0
Cork Oak	*Quercus suber*	0.06	0	0	0	0	0	0
King Palm Tree	*Archontophoenix alexandrae*	0.06	0	0	0	0	0	0
Orange Tree	*Citrus X sinensis*	0.07	0	0	0	0	0	0
Desert Willow	*Chilopsis linearis*	0.06	0	0	0.53	0	0.20	0
Afghan Pine	*Pinus eldarica*	0.07	0	0	0	0	0	0
Betula spp.	*Betula* spp.	0.05	0	0	0	0	0	0
Chinese Fringe Tree	*Chionanthus retusus*	0.05	0	0	0	0	0	0
Persian Silk Tree	*Albizia julibrissin*	0.08	0.32	0.37	0	0	0	0.23
Gleditsia spp.	*Gleditsia* spp.	0.05	0	0	0	0	0	0
Lemon Tree	*Citrus limon*	0.04	0	0	0	0	0	0
Morus spp.	*Morus* spp.	0.03	0	0	0	0	0	0
Pygmy Date Palm	*Phoenix roebelenii*	0.03	0	0	0	0	0	0
Coast Redwood	*Sequoia sempervirens*	0.04	0	0	0	0	0	0
Avocado Tree	*Persea americana*	0.02	0	0	0	0	0	0
Ginkgo	*Ginkgo biloba*	0.02	0	0	0	0	0	0
Japanese Maple	*Acer palmatum*	0.02	0	0	0	0	0	0

Majesty Palm	*Ravenea rivularis*	0.02	0	0	0	0	0	0
Acer spp.	*Acer* spp.	0.01	0	0	2.14	0	0.80	0
Alnus spp.	*Alnus* spp.	0.02	0	0	0	0	0	0
Apricot Tree	*Prunus* spp.	0.01	0	0	0	0	0	0
Cook Pine	*Araucaria columnaris*	0.02	0	0	0	0	0	0
Erythrina spp.	*Erythrina* spp.	0.19	0	0	0	0	0	0
Guadalupe Palm	*Brahea edulis*	0.02	0	0	0	0	0	0
Sago Palm	*Cycas revoluta*	0.01	0	0	0	0	0	0
Pencil Cactus	*Euphorbia tirucalli*	0.01	0	0	0	0	0	0
Peppermint Tree	*Agonis flexuosa*	0.01	0	0	0	0	0	0
Black Locust	*Robinia pseudoacacia*	0.02	0	0	0	0	0	0
Spanish Dagger Yucca	*Yucca gloriosa*	0.01	0	0	0	0	0	0
Monterey Pine	*Pinus radiata*	0.01	0	0	0	0	0	0
American Sycamore	*Prunus armeniaca*	0.01	0	0	0	0	0	0
Chinese Hibiscus	*Hibiscus rosa-sinensis*	0.01	0	0	0	0	0	0
Fountain Palm	*Livistona chinensis*	0.01	0	0	0	0	0	0
Red Tip Photinia	*Photinia × fraseri*	0.01	0	0	0	0	0	0
Golden Shower Tree	*Cassia fistula*	0.01	0	0	0	0	0	0
Guava Tree	*Psidium guajava*	0.01	0	0	0	0	0	0

Oleander	*Nerium oleander*	0.01	0	0	0	0	0	0
Peach Tree	*Prunus persica*	0.01	0	0	0	0	0	0
Pecan Tree	*Carya illinoinensis*	0.01	0	0	0	0	0	0
Star Magnolia	*Magnolia stellata*	0.01	0	0	0	0	0	0
Tilia spp.	*Tilia* spp.	0.01	0	0	0	0	0	0
Tree of Heaven	*Ailanthus altissima*	0.01	0	0	0	0	0	0

Table S5. Summary of migratory bird foraging behavior on nine common street-tree species in which we had sufficient foraging data (> 10 unique foraging observations that were > 30 sec in continuous observation). We display the number (*n*), total and mean observation time in seconds, the mean number of attacks and searches by migratory birds on each tree standardized per minute, and the attack index. The attack index – also referred to as the attack rate - is a ratio of attacks to searches, and is a measure of foraging success (Lovette and Holmes 1995, Oyugi et al. 2012, Wood et al. 2012).

Tree common name	*n*	Total observation (sec)	Mean observation (sec)	Attacks per min (mean)	Searches per min (mean)	Attack index
California Sycamore	20	1746	87.30	4.56	21.90	0.21
Camphor	20	1271	63.55	2.89	22.21	0.13
Carrotwood	40	2891	72.26	4.31	21.49	0.20
Chinese Elm	82	5996	73.12	4.14	20.46	0.20
Coast Live Oak	52	4639	89.21	4.62	21.58	0.21
Deodar Cedar	11	667	60.63	4.36	20.91	0.21
Holly Oak	14	1144	81.71	3.14	18.24	0.17
Italian Stone Pine	10	862	86.20	6.76	28.24	0.24
Southern Live Oak	27	2147	79.59	4.57	21.75	0.21

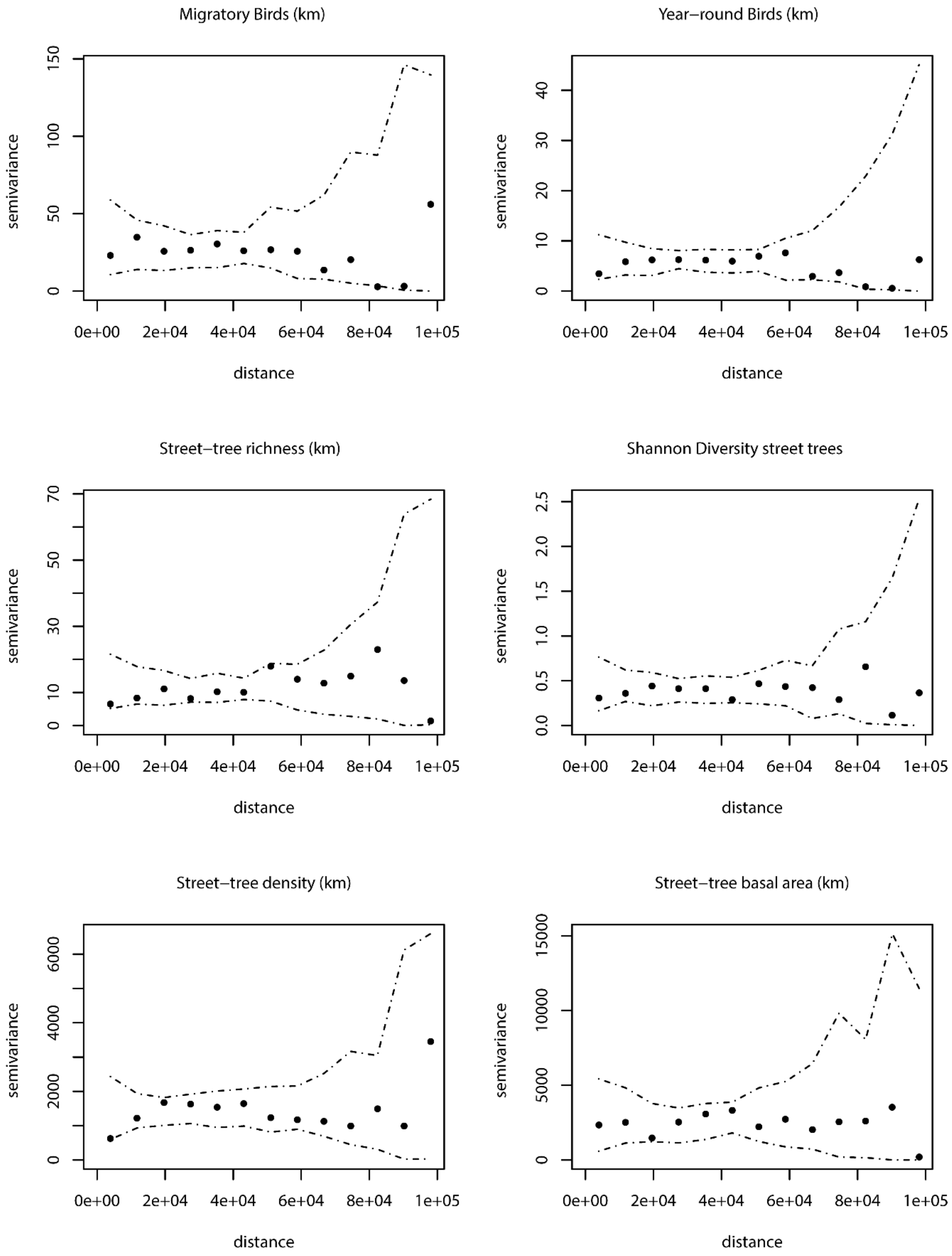
Migratory Birds (km)
Year–round Birds (km)
Street–tree richness (km)
Shannon Diversity street trees
Street–tree density (km)
Street–tree basal area (km)
semivariance
distance
0e+00
2e+04
4e+04
6e+04
8e+04
1e+05

Figure S1. Semivariograms of the residuals of the observed number of feeding migratory birds (km), the observed number of feeding year-round birds (km), street-tree richness (km), Shannon Diversity of street trees, street-tree density (km), and street-tree basal area m^2 (km) plotted as a function of the spatial arrangement of 36 survey locations. The semivariogram analysis bins residuals from residential survey locations that were similar and the semivariance at each lag is denoted as a black dot. The values on the x-axis are in meters. The dashed lines represent the maximum and minimum semivariances observed based on 99 random permutations of the original data. Semivariance values falling within the maximum and minimum semivariance envelopes indicate little evidence of spatial dependence of residuals among survey locations.

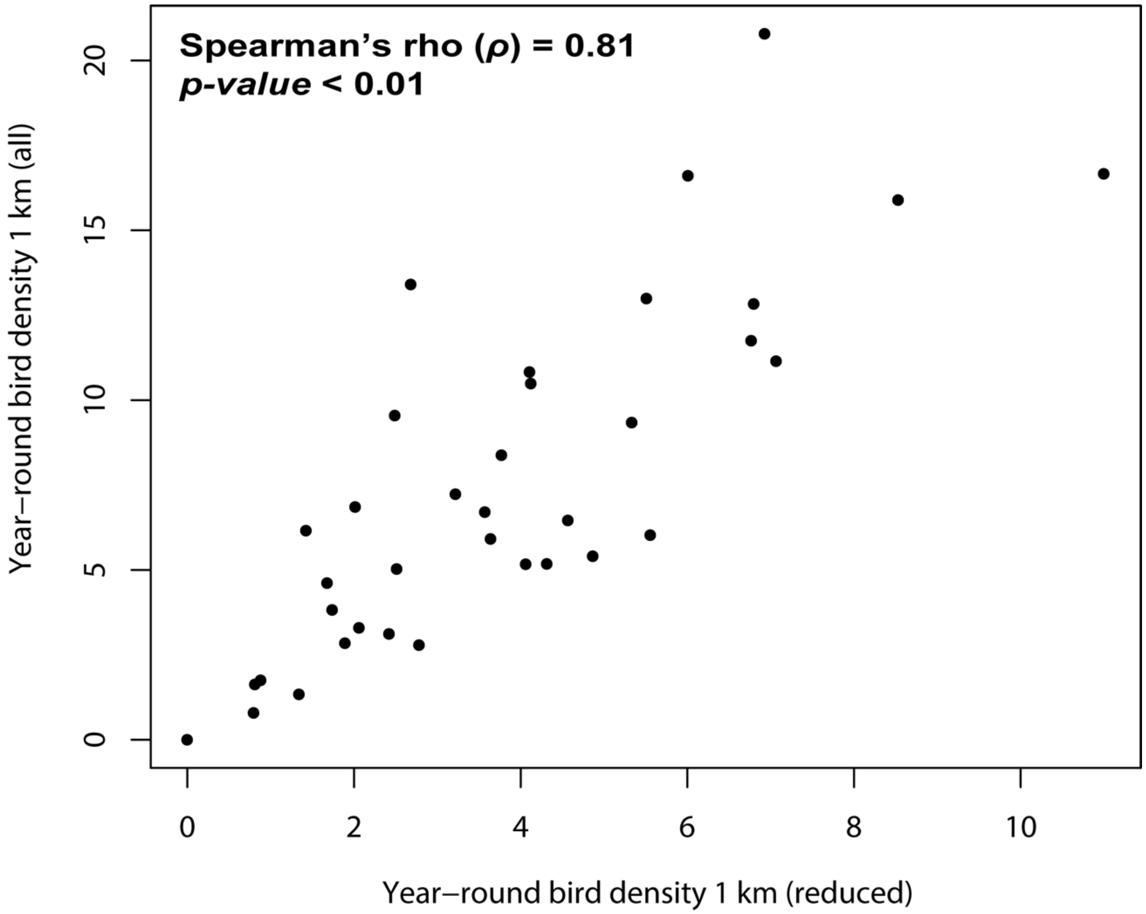

Figure S2. Scatterplot and associated Spearman's rho (ρ) correlation between 'reduced' and 'all' densities of year-round birds. 'Reduced' was a method where we treated all individuals of the same species within a large flock as an $n = 1$. We employed the reduced method to avoid overinflating the ecological importance of a given tree on the movement and feeding patterns of a group of birds. 'All' considered every individual bird within homogenous flocks. We standardized the tallies to 1-km of walking route, and thus refer to the numbers as density measures. The Spearman's ρ value of 0.81, *p-value* < 0.01 indicated a high correlation between both the reduced and all measures, suggesting both metrics yield similar effects in analyses.
